# Randomized Clinical Trial of Ringer’s Lactate Versus Chloride-Reduced Saline-Dextrose in Postoperative Kidney Transplantation

**DOI:** 10.1016/j.ekir.2026.107081

**Published:** 2026-09-17

**Authors:** Kornrawee Sawasdichai, Chanyanuch Rakpithayanon, Sirihatai Konwai, Thunyatorn Wuttiputhanun, Nuttha Lumlertgul, Kirada Apisutimaitri, Rattanaporn Burimsittichai, Athaya Vorasittha, Pea Pobpan, Adis Tasanarong, Asada Leelahavanichkul, Natavudh Townamchai, Kearkiat Praditpornsilpa, Somchai Eiam-Ong, Yingyos Avihingsanon, Suwasin Udomkarnjananun

**Affiliations:** 1 Division of Nephrology, Department of Medicine, Faculty of Medicine, Chulalongkorn University and King Chulalongkorn Memorial Hospital, Bangkok, Thailand; 2 Division of Nephrology, Department of Medicine, Faculty of Medicine, Prince of Songkla University, Songkla, Thailand; 3 Excellence Center for Solid Organ Transplantation, King Chulalongkorn Memorial Hospital, Thai Red Cross Society, Bangkok, Thailand; 4 Department of Anesthesiology, Faculty of Medicine, Chulalongkorn University and King Chulalongkorn Memorial Hospital, Bangkok, Thailand; 5 Department of Surgery, Faculty of Medicine, Chulalongkorn University and King Chulalongkorn Memorial Hospital, Bangkok, Thailand; 6 Division of Nephrology, Department of Internal Medicine, Faculty of Medicine, Thammasat University, Pathum Thani, Thailand; 7 Department of Microbiology, Center of Excellence on Translation Research in Inflammation and Immunology, Chulalongkorn University, Bangkok, Thailand; 8 Department of Microbiology, Faculty of Medicine, Chulalongkorn University and King Chulalongkorn Memorial Hospital, Bangkok, Thailand; 9 Department of Medicine, Faculty of Medicine, Center of Excellence Renal Immunology and Renal Transplantation, Chulalongkorn University, Bangkok, Thailand

**Keywords:** fluid management, kidney transplantation, normal saline solution, perioperation, postoperation, Ringer’s Lactate Solution

## Abstract

**Introduction:**

Normal saline administration induces hyperchloremic metabolic acidosis, potentially impairing kidney allograft recovery. The comparative efficacy of balanced crystalloids against actively chloride-reduced saline protocols remains unclear.

**Methods:**

This randomized, open-label trial compared Ringer’s Lactate Solution (RLS) with a dynamic dual-infusion protocol of normal saline and 5% dextrose (NSS + D5W) in adult kidney transplant recipients. Using a prespecified modified intention-to-treat analysis, 134 patients who received ≥2 L of study fluid were evaluated. Fluids were strictly titrated to hourly urine output. The primary outcome was day-4 serum bicarbonate. Secondary outcomes evaluated the overall treatment strategy, including protocolized rescue bicarbonate requirements, 1-month estimated glomerular filtration rate (eGFR) as an indicator of early functional recovery, and exploratory mechanistic biomarkers (urine neutrophil gelatinase-associated lipocalin [NGAL] and serum cytokines).

**Results:**

On postoperative day 4, the RLS group maintained significantly higher serum bicarbonate (19.39 vs. 17.31 mEq/L; *P* < 0.001) and required substantially less rescue bicarbonate therapy (17.9% vs. 58.2%; *P* < 0.001) compared with the NSS + D5W group. Serum chlorides were comparable between groups on day 4. RLS was associated with more favorable early allograft function, evidenced by a higher 1-month eGFR (61.63 vs. 53.18 ml/min per 1.73 m^2^; *P* = 0.028). Among the deceased-donor recipients, a lower proportion of RLS group had high urine NGAL (>100 ng/ml) and elevated interferon-γ; however, the continuous NGAL comparison was not significant and other cytokines (tumor necrosis factor [TNF]-α, interleukin [IL]-6, and IL-10) did not differ. These exploratory biomarker findings are hypothesis-generating.

**Conclusion:**

RLS effectively reduces early postoperative metabolic acidosis and was associated with better short-term allograft functional recovery compared with a chloride-reduced saline regimen. These benefits are most applicable to allografts exhibiting immediate or early function that permits standard volume expansion.

## Graphical Abstract

**Figure:**
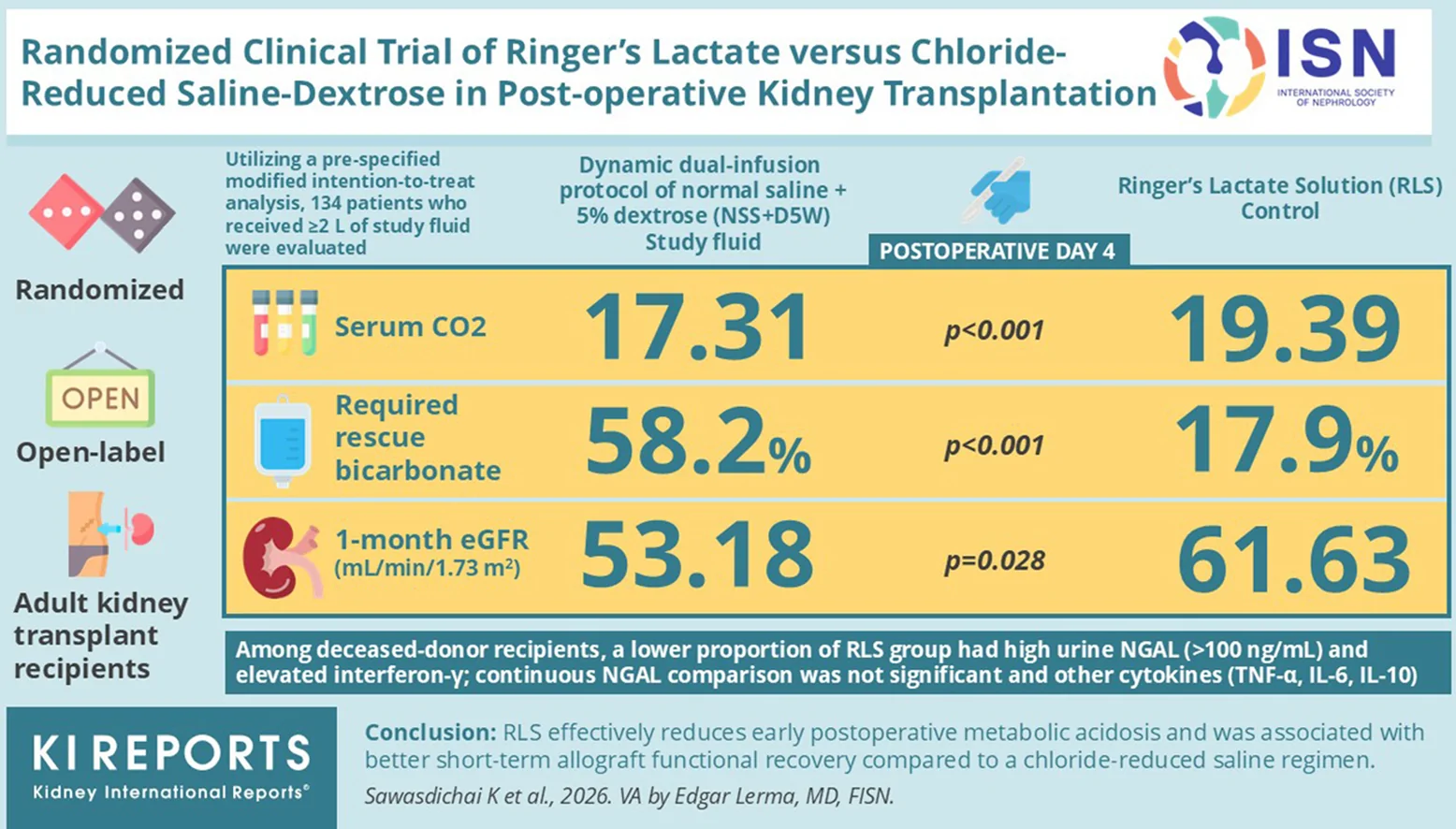

Kidney transplantation (KT) remains the most effective therapy for patients with end-stage kidney disease,^1^ but the immediate postoperative period is highly vulnerable. Deceased-donor allografts inevitably suffer ischemia-reperfusion injury (IRI)^2^ and frequently culminates in delayed graft function (DGF).^3^^,^^4^ Precise perioperative fluid management is therefore essential to maintain graft perfusion while avoiding electrolyte or acid-base derangements.

Although 0.9% normal saline is the historically standard resuscitation fluid,^5^ its unphysiological chloride content induces hyperchloremic metabolic acidosis, leading to renal vasoconstriction and acute kidney injury.^6^^,^^7^ Consequently, balanced crystalloids are increasingly preferred.^8^, ^9^, ^10^ BEST-Fluid trial^2^ reported that Plasma-Lyte-148 reduced DGF incidence compared with normal saline in deceased-donor KT (DDKT). However, pure saline delivers an unmitigated chloride load (154 mEq/L), potentially exaggerating the balanced solution’s relative benefit. Furthermore, proprietary fluids like Plasma-Lyte 148 are cost-prohibitive for resource-constrained settings, including low- or middle-income countries such as Thailand.

To mitigate chloride toxicity, many centers use a real-world dual-infusion strategy of NSS + D5W. It remains unknown whether balanced crystalloids retain superiority against this physiologically conscientious, chloride-reduced protocol. As an affordable and widely accessible alternative, RLS fulfills the need for a single-solution balanced crystalloid. This study evaluates the efficacy and safety of RLS versus an active NSS + D5W control in adult KT recipients. Beyond routine functional assessments, we mechanistically evaluated early allograft injury by quantifying systemic inflammatory cytokines and urine NGAL to comprehensively capture the physiological impact of these fluid strategies.

## Methods

### Study Overview and Study Population

This study was an open-label, investigator-initiated, randomized clinical trial conducted at King Chulalongkorn Memorial Hospital (Bangkok, Thailand). The trial protocol was registered with ClinicalTrials.gov (NCT06030609) and approved by the institutional review board of the Faculty of Medicine, Chulalongkorn University (IRB No. 281/67). Adult patients (age ≥18 years) undergoing first KT from either living or deceased donors were eligible for inclusion. Patients undergoing multiorgan transplantation, which requires different fluid strategy, were excluded.

### Randomization and Fluid Interventions

Enrolled participants were randomly assigned (1:1), stratified by donor type, to receive either RLS or NSS + D5W for postoperative i.v. fluid management. Fluid replacement rates were strictly titrated to hourly urine output. The control group received a dynamic dual-infusion (NSS + D5W) algorithm adjusted to output thresholds, whereas the intervention group received RLS as a single continuous 1:1 replacement infusion. The complete fluid titration algorithms are detailed in the Supplementary Table S1. Because i.v. fluid was titrated 1:1 to hourly urine output, the largest volumes were usually administered during the early diuretic phase on postoperative days 0 and 1, tapering thereafter as urine output declined and oral intake resumed in standard cases. Study fluids were discontinued once oral intake was adequate (typically by postoperative day 3–4), or in recipients who developed sustained oliguria, once the declining urine output no longer warranted matched replacement, after which hydration was maintained by oral intake to avoid volume overload. This urine-matched algorithm applied to all participants regardless of graft function, including those who developed DGF.

Clinical volume status was rigorously monitored (via vital signs, weights, and bedside ultrasonography) until study fluids were discontinued upon adequate oral intake. Severe metabolic acidosis (serum bicarbonate < 15 mEq/L) triggered a tiered rescue bicarbonate protocol, adapted from the BICAR-ICU trial^11^ (Supplementary Table S2). Rescue therapy was initiated based on each individual patient’s serum bicarbonate at any single daily measurement (the individual nadir) rather than on a group mean or an averaged value. Because severe acidosis could not ethically be left untreated, patients meeting the prespecified threshold received rescue alkali therapy; consequently, the reported daily group means represent on-treatment values and lie above the underlying individual nadir distribution. The full study protocol is provided in Supplementary Material.

### Immunosuppressive Protocol

Standard induction (basiliximab or antithymocyte globulin) and maintenance regimens (tacrolimus, mycophenolic acid, and corticosteroids) were used based on immunologic risk. Specific dosing, tapering schedules, and target trough concentrations are detailed in the Supplementary Table S1.

### Clinical and Biomarker Assessments

Postoperative laboratory parameters—including blood glucose, blood urea nitrogen, serum creatinine, electrolytes, and complete blood counts—were monitored daily per the trial protocol. Venous blood pH was assessed on postoperative day 4.

To evaluate early allograft injury and the IRI process, specific biomarker assays were performed on postoperative day 4 for consenting participants (requiring additional blood drawing). Day 4 was prespecified as the primary acid–base and biomarker assessment timepoint because study i.v. fluids are typically discontinued by day 3 to 4, allowing for the assessment of the cumulative fluid effect while minimizing interference from late postoperative confounders. Assessments included urine NGAL as a marker of acute tubular injury, and serum inflammatory cytokines (interferon [IFN]- γ, TNF-α, IL-6, and IL-10) using multiplex assays (BioLegend, San Diego, CA).

Graft function was evaluated at 1 week and 1 month after transplantation using the eGFR rate (Chronic Kidney Disease-Epidemiology Collaboration eGFR) and 24-hour urine creatinine clearance.

### Outcomes

The primary outcome was the incidence and severity of metabolic acidosis, quantified by the serum bicarbonate concentration on postoperative day 4. Secondary outcomes included longitudinal postoperative electrolyte and blood glucose trends, the incidence of DGF, and early graft function parameters (serum creatinine and 24-hour creatinine clearance at 1 week and 1 month). Additional prespecified secondary outcomes included the day 4 biomarker profiles (urine NGAL and serum cytokines). DGF was defined as the requirement for dialysis within the first 7 postoperative days for any indication (refractory hyperkalemia, volume overload unresponsive to diuretics, or uremia); dialysis was initiated at the discretion of the attending nephrologist. Because this definition rests on the requirement for dialysis rather than on urine output, it also captured nonoliguric DGF—recipients with adequate urine output but insufficient solute clearance—and recipients whose graft function was initially adequate but declined thereafter.

Because severe metabolic acidosis triggered protocolized rescue bicarbonate therapy, the target for our analysis evaluates the effect of the overall randomized treatment strategy (the assigned fluid protocol plus prespecified usual rescue care), rather than the direct physiological effect of the assigned fluid in the absence of rescue therapy. By strictly protocolizing the rescue criteria (serum bicarbonate < 15 mEq/L), we sought to minimize the performance bias inherent to an open-label design study.

### Statistical Analysis

Sample size calculations were based on the primary outcome of day 4 serum bicarbonate levels. Assuming a SD of 5.0 mEq/L, a conservative estimate derived from previous deceased-donor trials^12^ to account for a heterogeneous donor population, 63 patients per arm were required to detect a clinically meaningful difference of 2.5 mEq/L with 80% power and a 2-sided alpha of 0.05. To account for an anticipated 10% exclusion rate required to meet the modified intention-to-treat (mITT) criteria (which predefined exclusion for patients receiving an insufficient study fluid volume of <2 L), a minimum of 140 patients was required.

The primary analysis followed a mITT principle, restricting the cohort to randomized transplant recipients who received ≥2 L of study fluid. This *a priori* threshold ensured sufficient ionic exposure to assess biochemical outcomes, minimizing dilution bias from unexposed patients. Patients receiving <2 L were excluded because of immediate postoperative oligo-anuria requiring strict fluid restriction. Because this anuria occurred before meaningful fluid exposure, the exclusion reflects baseline physiology rather than a treatment-induced bias. This mITT framework and the <2 L exclusion were prespecified in the trial protocol and registered at ClinicalTrials.gov (NCT06030609), before the onset of recruitment; no *post hoc* modifications to the analysis population or outcomes were made.

Baseline characteristics were summarized using means (SD) for normally distributed continuous variables, medians with interquartile ranges (IQR) for skewed data, and frequencies with percentages for categorical variables. Intergroup comparisons at single time points used unpaired *t* tests, Wilcoxon rank-sum tests, or chi-square tests, as appropriate. Longitudinal treatment effects across multiple time points were analyzed using multilevel mixed-effects linear regression. All statistical analyses were performed using STATA version 19 (StataCorp, College Station, TX), with a 2-sided *P*-value of <0.05 indicating statistical significance.

## Results

### Baseline Characteristics

Between September 2024 and March 2026, a total of 160 patients were assessed for eligibility. One patient was excluded before randomization because of a planned multiorgan transplant. The remaining 159 patients were stratified and randomly assigned to receive either the active control (NSS + D5W, *n* = 79) or the intervention (RLS, *n* = 80). After randomization, 25 patients were excluded from the mITT analysis, because they received a cumulative study fluid volume of less than 2 L. Consequently, 134 patients (67 in each arm) completed the 1-month trial follow-up and were included in the final analyses (Figure 1).

**Figure 1:**
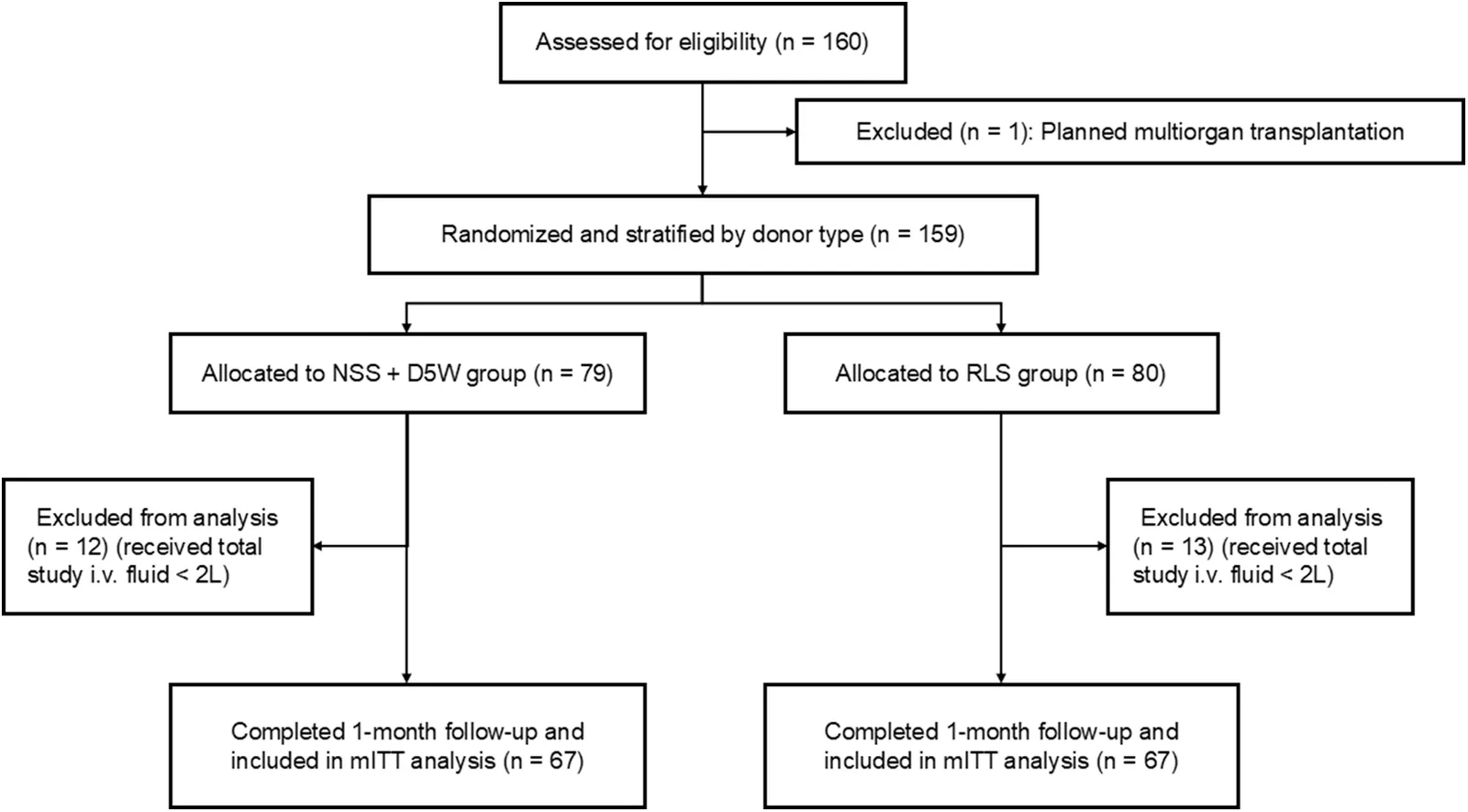
CONSORT flow diagram of study participants. CONSORT, Consolidated Standards of Reporting Trials; mITT, modified intention-to-treat; NSS + D5W, normal saline and 5% dextrose; RLS, Ringer’s Lactate Solution.

Baseline characteristics were well-balanced between the 2 groups (Table 1). The cohort was predominantly male, and 68.7% received DDKT.

**Table 1:** Baseline characteristics of study population

Variables	RLS (*n* = 67)	NSS + D5W (*n* = 67)
Male, *n* (%)	35 (52.2)	44 (65.7)
Age, yrs, mean (SD)	46.40 (13.08)	45.43 (13.58)
Dialysis vintage, yrs, mean (SD)	4.57 (2.82)	4.47 (2.82)
Mode of KRT, *n* (%)		
HD	56 (83.6)	60 (89.6)
PD	7 (10.5)	4 (6.0)
Preemptive	4 (6.0)	3 (4.5)
DDKT, *n* (%)	46 (68.7)	46 (68.7)
ABOi, *n* (%)	5 (7.5)	7 (10.5)
HLA MM, mean (SD)	3.06 (1.85)	3.48 (1.82)
% PRA, median (IQR)	0 (0-0) (range 0–97)	0 (0-0) (range 0–100)
Diabetes mellitus, *n* (%)	14 (20.9)	13 (19.4)
KDPI, median (IQR)	30 (11–44)	27 (9–54)
DDKT KDPI	40 (24–54)	42 (23–60)
LDKT KDPI	0 (−6 to 23)	2 (−7 to 15)
Induction, *n* (%)		
ATG	4 (6.0)	8 (11.9)
Basiliximab	63 (94.0)	59 (88.1)
Total ischemic time, hours, mean (SD)		
DDKT	18.74 (3.56)	18.57 (4.52)
LDKT	1.18 (0.29)	1.11 (0.29)

ABOi, ABO incompatible; ATG, anti-thymocyte globulin; DDKT, deceased donor kidney transplantation; HD, hemodialysis; HLA MM, human leukocyte antigen mismatch; KDPI, kidney donor profile index; KRT, kidney replacement therapy; LDKT, living donor kidney transplant; NSS + D5W, normal saline solution and 5% dextrose water; PD, peritoneal dialysis; PRA, panel reactive antibodies; RLS, Ringer’s lactate solution.

### I.v. Fluid Administration

The volumes of protocol-directed and nonprotocol i.v. fluids are summarized in Supplementary Table S3. The mean total volume of protocol i.v. fluid administered over the study period did not differ significantly between the groups (9521.57 [4726.12] mL in RLS group vs. 9944.52 [4723.03] mL in NSS + D5W group; *P* = 0.61). In the majority of patients, study i.v. fluids were successfully discontinued by postoperative day 4 (118 of 134 patients; 88%); the remaining 16 stopped i.v. fluids on day 5. Subgroup analyses by donor type showed similar fluid requirements, with no significant volumetric differences observed in either DDKT recipients or living donor kidney transplant (LDKT) recipients. Daily i.v. fluid volumes remained balanced between arms: approximately 70% of the total protocol fluid was administered within the first 48 hours (RLS: 6760 ml of 9,522 ml; NSS + D5W: 6960 ml of 9945 ml), approximately 89% by the end of day 2, and more than 99% by the end of day 4, after which daily volumes fell below 300 ml (Supplementary Figure S1A and B; Supplementary Table S4).

### Primary Outcome: Serum Bicarbonate and Acid-Base Status

Preoperative baseline (day 0) serum bicarbonate levels were comparable between the RLS and NSS + D5W groups (23.52 [3.52] vs. 24.15 [4.03] mEq/L; *P* = 0.34). After surgery and continuous fluid administration, a progressive decline in bicarbonate levels was observed universally; however, the rate of decline was significantly attenuated in the RLS group (Figure 2). The slope of bicarbonate reduction was −0.91 mEq/L/d (95% CI: -1.11 to -0.72) in the RLS group compared with -1.60 mEq/L/d (95% CI: -1.80 to -1.41) in the NSS + D5W group, representing a statistically significant slope difference of 0.69 mEq/L/day (95% CI: 0.41–0.97; *P* < 0.001).

**Figure 2:**
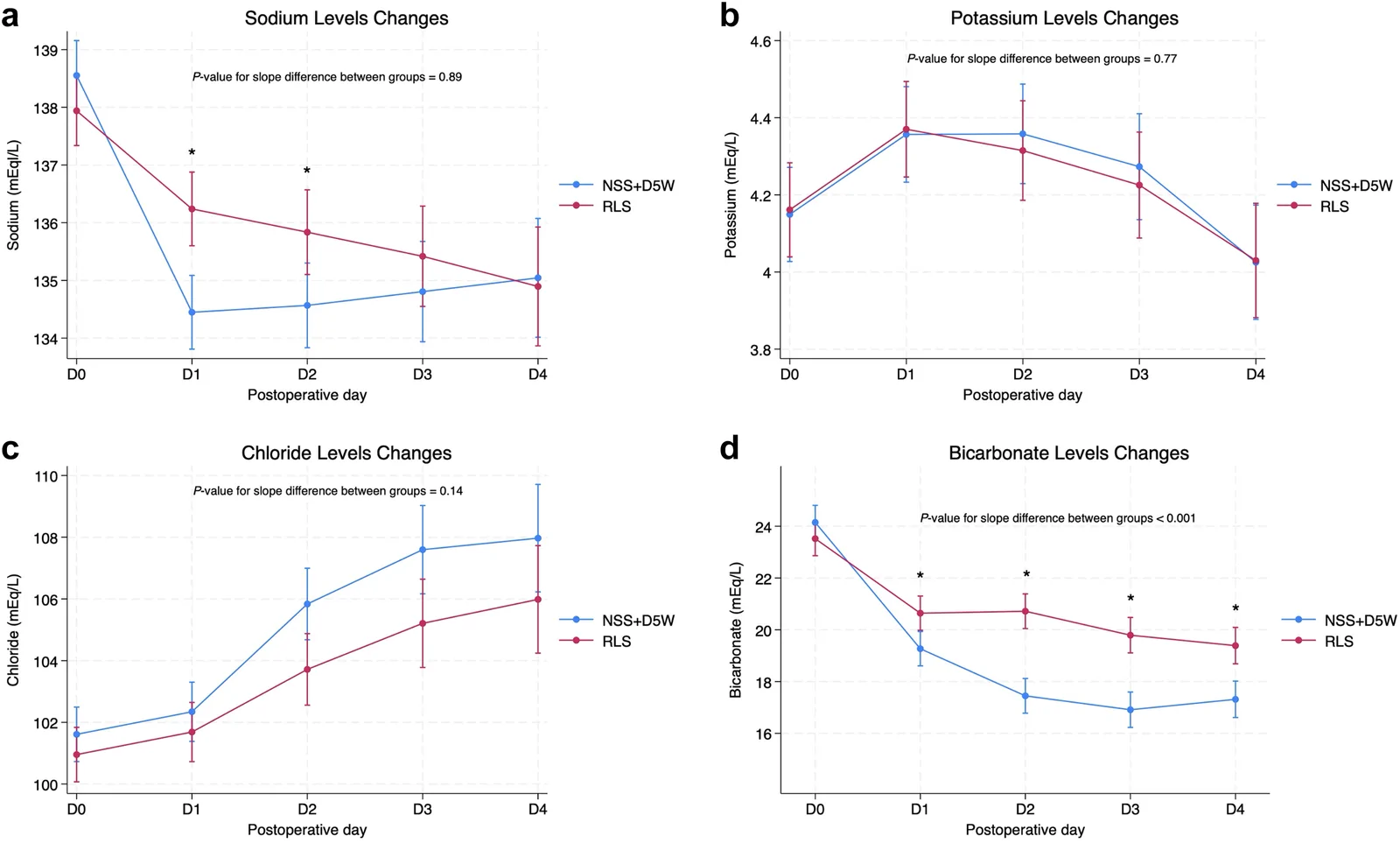
Longitudinal changes in serum electrolyte concentrations during the early postoperative period. Postoperative trajectories of (a) serum sodium, (b) serum potassium, (c) serum chloride, and (d) serum bicarbonate from day 0 (baseline) to postoperative day 4. Asterisks indicate a statistically significant difference (*P* < 0.05) between the two groups at that specific time point. Data are presented as mean values; error bars represent 95% CI. CI, confidence interval; NSS + D5W, normal saline and 5% dextrose; RLS, Ringer’s Lactate Solution.

Consequently, for the primary outcome on postoperative day 4, the RLS group maintained significantly higher serum bicarbonate levels compared with the active control (19.39 [2.50] mEq/L vs. 17.31 [2.15] mEq/L; *P* < 0.001). Venous pH was correspondingly higher in the RLS group (7.41 [0.06] vs. 7.37 [0.07]; *P* = 0.013), and the proportion of patients developing clinical metabolic acidosis (pH < 7.35) was significantly lower (Table 2). In an exploratory subgroup analysis, these primary-outcome differences appeared predominantly in the DDKT cohort. Other serum electrolytes, including sodium, potassium, and chloride, exhibited no significant differences in their day 4 concentrations. The longitudinal postoperative trends between the groups are demonstrated in Supplementary Table S5 and Supplementary Figures S2 and S3. Serum chloride was modestly but significantly lower in the RLS group on days 2 and 3 (day 2: 103.72 vs. 105.84 mEq/L, *P* = 0.02; day 3: 105.21 vs. 107.60 mEq/L, *P* = 0.03).

**Table 2:** Primary metabolic acidosis and secondary allograft outcomes by i.v. fluid regimen and donor type

Overall	RLS (*n* = 67)	NSS + D5W (*n* = 67)	*P-*value
HCO_3_ day4, mEq/L, mean (SD)	19.39 (2.50)	17.31 (2.15)	<0.001
pH day4, mean (SD)	7.41 (0.06)	7.37 (0.07)	0.013
pH < 7.35, *n* (%)	4 (5.9)	16 (23.8)	0.012
DGF, *n* (%)	2 (3.0)	6 (9.0)	0.14
SCr day 7, mg/dl, mean (SD)	2.59 (2.39)	2.93 (2.11)	0.39
At 1 mo post KT			
SCr, mg/dl, mean (SD)	1.37 (0.53)	1.63 (0.66)	0.01
eGFR CKD-EPI, ml/min per 1.73 m^2^, mean (SD)	61.63 (22.70)	53.18 (21.37)	0.028
24 h-urine CCr, ml/min, mean (SD)	54.03 (18.23)	48.26 (16.68)	0.06

**DDKT**	**RLS (n = 46)**	**NSS + D5W (n = 46)**	***P*****value**

HCO_3_ day 4, mEq/L, mean (SD)	19.83 (2.56)	17.30 (2.15)	< 0.001
pH day 4, mean (SD)	7.42 (0.04)	7.37 (0.08)	< 0.001
pH < 7.35, *n* (%)	2 (4.3)	14 (30.4)	0.004
DGF, n (%)	2 (4.4)	5 (10.9)	0.24
SCr day 7, mg/dl, mean (SD)	3.21 (2.66)	3.60 (2.22)	0.81
At 1 mo post KT			
SCr, mg/dl, mean (SD)	1.47 (0.58)	1.78 (0.74)	0.027
eGFR CKD-EPI, ml/min per 1.73 m^2^, mean (SD)	58.32 (24.37)	48.58 (22.38)	0.049
24 h-urine CCr, ml/min, mean (SD)	51.68 (19.30)	43.30 (16.31)	0.028

**LDKT**	**RLS (*****n*****= 21)**	**NSS + D5W (*****n*****= 21)**	***P-*****value**

HCO_3_ day 4, mEq/L, mean (SD)	18.43 (2.11)	17.33 ± 2.20	0.11
pH day 4, mean (SD)	7.37 (0.08)	7.39 ± 0.04	0.47
pH < 7.35, *n* (%)	2 (9.5)	2 (9.5)	0.99
DGF, *n* (%)	0 (0)	1 (4.8)	0.10
SCr day 7, mg/dl, mean (SD)	1.22 (0.28)	1.46 (0.55)	0.07
At 1 mo post KT			
SCr, mg/dl, mean (SD)	1.15 (0.28)	1.32 (0.27)	0.05
eGFR CKD-EPI, ml/min per 1.73 m^2^, mean (SD)	68.90 (16.83)	63.23 (14.99)	0.26
24 h-urine CCr, ml/min, mean (SD)	58.96 (15.01)	59.12 (11.80)	0.97

24hr-urine CCr, 24 hours urine creatinine clearance; DDKT, deceased donor kidney transplantation, DGF, delayed graft function; eGFR-EPI, estimated glomerular filtration rate using the Chronic Kidney Disease Epidemiology Collaboration equation; HCO_3_, serum bicarbonate level; LDKT, living donor kidney transplant; NSS + D5W, normal saline solution and 5% dextrose water; RLS, Ringer’s lactate solution; SCr, serum creatinine.

### Clinical Secondary Outcomes: Kidney Allograft Function

The incidence of DGF was numerically lower in the RLS group (3.0%) compared with the NSS + D5W group (9.0%), though this difference did not reach statistical significance (*P* = 0.14; Table 2). Early graft function, assessed by serum creatinine on postoperative day 7, was comparable between the RLS and NSS + D5W groups. The slopes of serum creatinine and eGFR changes during the first postoperative week did not differ significantly for the overall cohort (Figure 3).

**Figure 3:**
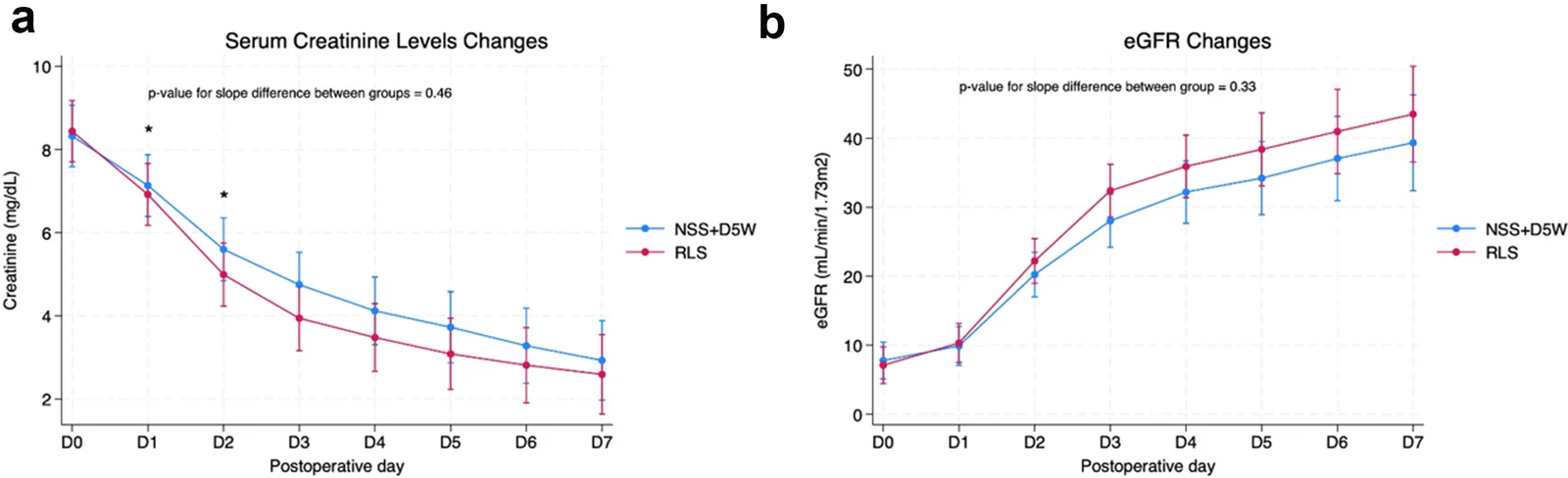
Longitudinal changes in kidney allograft function during the first postoperative week. Postoperative trajectories of (a) serum creatinine and (b) estimated glomerular filtration rate (eGFR) from day 0 (baseline) to postoperative day 7. Asterisks indicate a statistically significant difference (*P* < 0.05) between the two groups at that specific time point. Data are presented as mean values; error bars represent 95% CI. CI, confidence interval; NSS + D5W, normal saline and 5% dextrose; RLS, Ringer’s Lactate Solution.

However, at 1-month post-transplantation, the RLS group demonstrated a significantly higher eGFR compared with the NSS + D5W group (61.63 [22.70] vs. 53.18 [21.37] ml/min per 1.73 m^2^; *P* = 0.028), and mean serum creatinine levels were lower (1.37 [0.53] vs. 1.63 [0.66] mg/dl; *P* = 0.01; Table 2). The 24-hour urine creatinine clearance at 1 month also trended higher in the RLS group (54.03 [18.23] vs. 48.26 [16.68] ml/min; *P* = 0.06). In a prespecified subgroup analysis of DDKT recipients, the RLS group exhibited a significantly faster rate of serum creatinine decline (slope difference: −0.11 mg/dl/d; *P* = 0.042) and a more robust daily increase in eGFR (slope difference: 0.78 ml/min per 1.73 m^2^/d; *P* = 0.028) compared with the NSS + D5W group (Supplementary Figures S4 and S5). In the exploratory, prespecified subgroup analyses, the 1-month functional differences appeared predominantly in the DDKT subgroup, in which RLS was associated with lower 1-month serum creatinine, higher eGFR, and higher 24-hour creatinine clearance (all *P* <0.05; Table 2).

### Mechanistic Secondary Outcomes: Tubular Injury Markers and Inflammatory Cytokines

To evaluate the impact of fluid choice on early tubular injury, day 4 urine NGAL was quantified in a subset of consenting recipients. Among DDKT recipients, median urine NGAL levels were numerically lower in the RLS group (55.5 [31–101.63] ng/ml) compared with the NSS + D5W group (122.45 [96.34–177.07] ng/ml), though the continuous variable comparison was not statistically significant (*P* = 0.18; Figure 4). However, the proportion of DDKT recipients exhibiting high tubular injury (defined categorically as urine NGAL > 100 ng/mL) was significantly reduced in the RLS group (28.6% vs. 52.9%; *P* = 0.039; Table 3). No significant differences in urine NGAL parameters were observed among LDKT recipients. This categorical threshold analysis (urine NGAL > 100 ng/ml) was exploratory, and the biomarker findings should be interpreted as hypothesis-generating rather than as definitive evidence of reduced tubular injury.

**Figure 4:**
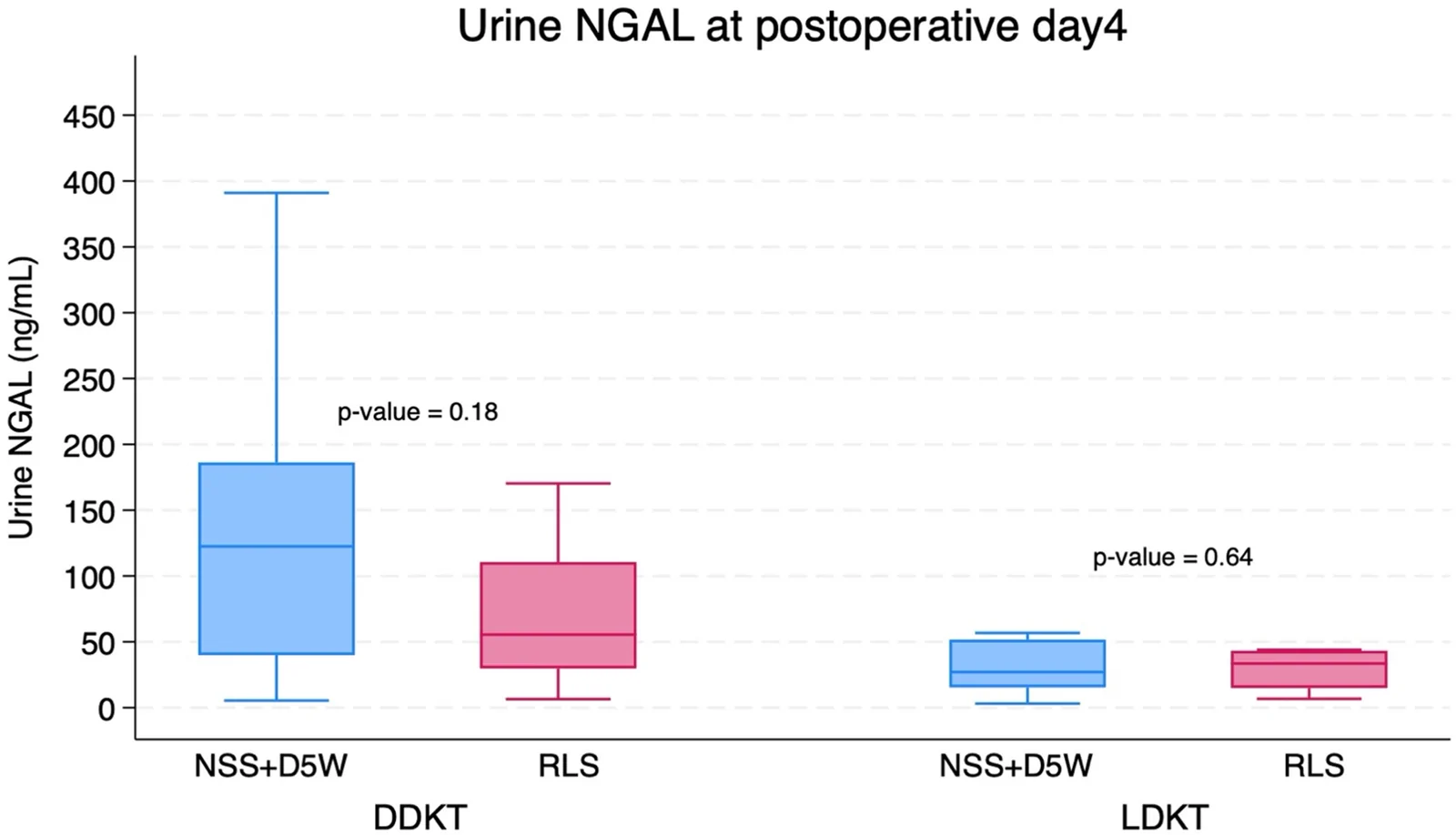
Urine neutrophil gelatinase-associated lipocalin (NGAL) concentrations on postoperative day 4 stratified by donor type. Box plot illustrating the distribution of urine NGAL levels in the active control (NSS + D5W) and intervention (RLS) groups among deceased donor kidney transplant (DDKT) and living donor kidney transplant (LDKT) recipients. The horizontal line within the box represents the median, the box bounds indicate the interquartile range, and the whiskers extend to the minimum and maximum observed values. NSS + D5W, normal saline and 5% dextrose; RLS, Ringer’s Lactate Solution.

**Table 3:** Proportion of patients with elevated markers of tubular injury and systemic inflammation on postoperative day 4

Variables	RLS	NSS + D5W	*P-*value
High urine NGAL > 100 ng/ml, *n*/total (%)			
DDKT	10/35 (28.6)	18/34 (52.9)	0.039
LDKT	2/15 (13.3%)	3/16 (18.8)	0.68
High IFN-γ (> 4.92 pg/ml), *n*/total (%)			
DDKT	9/27 (33.3)	18/30 (60.0)	0.044
LDKT	7/13 (53.9)	6/10 (60.0)	0.77
High TNF-α (> 7.98 pg/ml), *n*/total (%)			
DDKT	15/27 (55.6)	16/30 (53.3)	0.86
LDKT	6/13 (46.2)	3/10 (33.3)	0.43
High IL-6 (> 29.29 pg/ml), *n*/total (%)			
DDKT	17/27 (63.0)	13/30 (43.3)	0.14
LDKT	6/13 (46.2)	4/10 (40.0)	0.77
High IL-10 (> 15.95 pg/ml), %			
DDKT	16/27 (59.3)	16/30 (53.3)	0.65
LDKT	5/13 (38.5)	3/10 (30.0)	0.67

DDKT, deceased donor kidney transplantation; IFN-γ, interferon gamma; IL-6, interleukin-6; IL-10, interleukin-10; LDKT, living donor kidney transplant; NGAL, neutrophil gelatinase associated lipocalin; NSS + D5W, normal saline solution and 5% dextrose water; RLS, Ringer’s lactate solution; TNF-α, tumor necrotic factor-alpha.

High serum cytokine concentrations were defined using the median cohort values as cutoffs.

Systemic inflammatory profiling on postoperative day 4 revealed that a significantly lower proportion of patients in the RLS group had elevated IFN-γ levels (defined as > median study concentration) compared with the active control (33.3% vs. 60.0%; *P* = 0.044). Concentrations of other measured cytokines, including TNF-α, IL-6, and IL-10, did not differ significantly between the treatment arms (Table 3; Supplementary Figure S6).

### Adverse Events and Rescue Therapies

The requirement for protocol-directed rescue bicarbonate therapy was drastically reduced in the RLS arm; only 17.9% of RLS patients required intervention compared with 58.2% in the NSS + D5W group (*P* < 0.001; Supplementary Figure S7A; Supplementary Table S6). Among those who met the threshold for rescue therapy, the total dosage of oral sodium bicarbonate required over the first 5 days was comparable between groups (Supplementary Figure S7B).

The incidence of hyperkalemia, significant hyperglycemia, and hemodynamic instability, as well as the requirement for potassium-binding resins, were low and comparable between groups (Supplementary Figure S8; Supplementary Table S6).

## Discussion

This randomized controlled trial demonstrates that RLS is superior to a physiologically guided, reduced-chloride control (NSS + D5W) in maintaining acid-base homeostasis and is associated with better preservation of early allograft function. Although previous studies largely focused on only intraoperative fluid choices,^13^^,^^14^ this investigation evaluated postoperative fluid strategies extending beyond the operating room and continued until oral intake resumed (typically by day 3–4). RLS effectively mitigated metabolic acidosis, drastically reduced rescue bicarbonate requirement, and yielded a higher 1-month eGFR. In exploratory analyses, RLS was associated with a lower proportion of DDKT recipients showing high urine NGAL and elevated IFN-γ; however, the continuous NGAL comparison was not significant and TNF-α, IL-6, and IL-10 did not differ, so these findings are hypothesis-generating.

Serum bicarbonate trajectories revealed a significantly steeper decline in the NSS + D5W group. The metabolic superiority of RLS aligns with physiological studies highlighting lactate as an effective circulating buffer upon hepatic metabolism.^15^ Although the NSS + D5W dual-infusion protocol used in this trial is not universally adopted, some centers employ this strategy as their local or regional standard of care to avoid chloride-rich fluids. Consequently, the selection of this active control serves as a unique methodological strength. Traditional trials evaluating perioperative fluids often compare balanced crystalloids against pure normal saline, a design that inevitably delivers an unmitigated chloride load and may consequently exaggerate the relative benefits of the balanced solution. In contrast, both fluid protocols used in our trial limited the overall infused chloride burden, largely avoiding postoperative hyperchloremia across both groups. Therefore, the metabolic advantage of RLS observed here is unlikely to be solely attributed to the avoidance of hyperchloremic-induced renal vasoconstriction.^16^, ^17^, ^18^ Instead, by isolating the intrinsic impact of the lactate buffer from the confounding effects of severe hyperchloremia, our findings suggest that the lactate buffer provides an independent metabolic advantage. This emphasizes that the qualitative composition of a fluid—specifically the presence of a circulating buffer, and not merely its chloride restriction or total volume—may dictate early metabolic stability. Although serum chloride was modestly lower in the RLS arm on days 2 and 3, both groups remained normochloremic, and a difference of approximately 2 mEq/L within the normal range is unlikely to account for the observed benefits; a minor chloride-mediated contribution nonetheless cannot be entirely excluded.

Because fluid was matched to urine output, roughly 70% of the assigned solution was delivered within the first 48 hours. Importantly, the following between-group divergence in acid–base status emerged during this exposure window and persisted after fluids were withdrawn: serum bicarbonate already differed on day 1 (20.64 vs. 19.27 mEq/L; *P* = 0.006) and day 2 (20.72 vs. 17.45 mEq/L; *P* < 0.001), and the difference remained significant on day 4 (19.39 vs. 17.31 mEq/L; *P* < 0.001), by which time more than 99% of study fluid had been administered and infusions had effectively ceased. This temporal pattern is mechanistically coherent, as the first 48 hours encompass reperfusion and the peak of ischemia–reperfusion injury—precisely the window in which the composition of the infused fluid would be expected to matter most. Rather than limiting the findings, this suggests that a brief, front-loaded exposure during the vulnerable early period might produce a durable metabolic difference that persisted beyond the infusion itself and was associated with improved functional recovery at 1 month.

In exploratory subgroup analyses, treatment responses appeared to differ by donor type, with the apparent benefits of RLS concentrated in the DDKT cohort; given the limited subgroup sizes, these observations are hypothesis-generating. DDKT pathophysiology involves obligate, profound IRI exacerbated by cold ischemia and brain-death inflammatory cascades.^19^^,^^20^ These heavily stressed tubules are uniquely vulnerable to secondary insults like metabolic acidosis, which aggravates cellular injury^21^^,^^22^ and propagate tubular stress.^6^^,^^23^^,^^24^ By providing better acid-base control, RLS may have reduced secondary injury in these vulnerable allografts, a hypothesis supported by the lower proportion of recipients with high day-4 urine NGAL (>100 ng/mL). Similarly, a lower proportion of RLS recipients had elevated IFN-γ, which may reflect attenuated early inflammation.^25^ Beyond buffering, lactate may exert direct immunomodulatory effects by activating G-protein-coupled receptor 81, actively suppressing IRI-associated sterile inflammation.^26^

The lack of statistical difference in other measured cytokines (IL-6, IL-10, TNF-α) by day 4 likely reflect their divergent expression kinetics. Acute markers (TNF-α, IL-6) peak within hours of reperfusion,^27^^,^^28^ whereas regulatory cytokines (IL-10) orchestrate later repair phases.^29^^,^^30^ In contrast, IFN-γ—which bridges the innate and adaptive alloimmune responses—typically reaches its biological peak between postoperative days 3 and 5, aligning with the day-4 sampling window used in this study.^31^^,^^32^ Any sustained secondary inflammatory cascades were likely blunted by the robust induction immunosuppression. A notable limitation of the current study is the reliance on a single biomarker assessment on postoperative day 4. Although serial daily measurements would more accurately capture these complex, biphasic kinetic trends, such dense sampling was precluded by the pragmatic design of the trial, resource constraints associated with multiplex assays, and the clinical intent to minimize early postoperative phlebotomy. Future studies incorporating longitudinal cytokine tracking are warranted to fully elucidate the immunomodulatory timeline of specific buffer solutions following allograft reperfusion.

By using a mITT framework restricted to patients receiving ≥2 L of study fluid, this trial isolates the physiological impact of balanced crystalloids in allografts not limited by immediate oligo-anuria. In this population, RLS significantly reduced hyperchloremic acidosis and was associated with exploratory signals of attenuated IRI (a lower proportion of recipients with high urine NGAL and with elevated IFN-γ), although the continuous biomarker comparisons were not statistically significant. Notably, these results provide a counterpoint to the BEST-Fluids trial,^2^ which found a reduction in DGF but no difference in early eGFR. Because our total fluid received threshold inherently excluded the oligo-anuric patients most prone to DGF, our study was not positioned to show a difference in DGF rates. Instead, our findings show that in allografts with immediate or early function—excluding those strictly limited by severe oligo-anuria—adequate fluid volumes allow the metabolic benefits of RLS, together with exploratory molecular signals, to be accompanied by more favorable early clinical recovery, with a significantly higher 1-month eGFR that serves as a short-term indicator of functional recovery and may reflect the resolution of acute IRI.

These findings provide essential context when compared with the landmark BEST-Fluids trial,^2^ which reported the superiority of Plasma-Lyte-148 over normal saline in DDKT recipients. Although the present trial features a smaller sample size, it contributes unique perspectives to the literature. First, the observation period for laboratory assessment was extended until the cessation of i.v. fluids, compared with the 48-hour laboratory measures window in BEST-Fluids. Second, using NSS + D5W as an active control successfully isolated the impact of the buffer from the confounding effects of severe hyperchloremia. Third, incorporating DDKT and LDKT cohorts allowed for the exploratory comparison of fluid effects across different severities of IRI. Fourth, the integration of mechanistic biomarkers—specifically urine NGAL and systemic inflammatory cytokines— offers preliminary, hypothesis-generating insights into how fluid composition may relate to early tissue-level injury and immunomodulation. Finally, the single-center design ensured strict adherence to a homogenous, standardized protocol without the need for complex data imputation as shown in BEST-Fluids study.^2^

Economically, proprietary balanced solutions like Plasma-Lyte-148 are 5 to 10 times more expensive than RLS in Thailand. Given the high fluid volume required postoperatively, RLS emerges as a highly cost-effective alternative for resource-constrained systems. Furthermore, single-infusion RLS simplifies nursing workflows, reducing titration errors inherent to dual-infusion setups like NSS + D5W.

Several limitations warrant acknowledgment. The single-center design and sample size may restrict broad generalizability, although this remains 1 of the largest randomized controlled trials evaluating postoperative fluids in KT.^2^^,^^14^^,^^33^ Moreover, although the parallel inclusion of both living and deceased donor allografts provides a unique comparative analysis of fluid dynamics across different severities of IRI, these prespecified subgroups—particularly the smaller LDKT cohort—were not individually powered. Consequently, the lack of statistical significance in certain outcomes among LDKT recipients may be susceptible to type II error and should be interpreted as exploratory. Equally, the positive findings within the DDKT subgroup derived from prespecified but individually underpowered analyses, and should be regarded as exploratory and hypothesis-generating rather than confirmatory. Second, mechanistic biomarker data (urine NGAL and serum cytokines) were obtained in a subset of participants (75% and 60%, respectively), though missingness was balanced between the randomization arms. Third, the predefined mITT analysis excluded patients receiving <2 L of study fluid; because fluid was titrated 1:1 to urine output, this postrandomization exclusion identifies recipients with immediate oligo-anuria in whom the intervention was effectively never delivered, rather than an outcome-driven selection. Exclusions were balanced between arms (13 RLS vs. 12 NSS + D5W); nonetheless, the benefits of RLS in this study are consequently most applicable to allografts with immediate or early function and may not extend to recipients with severe initial oliguria, and a full intention-to-treat analysis was neither prespecified nor feasible, as the outcomes measurement were complete only within the mITT population. Because some recipients at DGF risk were among those excluded (immediate oligo-anuria), the DGF incidence reported here reflects the analyzed cohort rather than the real-world rate. Fourth, serum lactate was not measured systematically, so cohort-wide lactate values and the incidence of hyperlactatemia cannot be reported, although the lactate load delivered by RLS (28 mEq/L) is modest and is rapidly metabolized by the liver to bicarbonate in patients with intact hepatic function; no case of hyperlactatemia or lactic acidosis was clinically observed. Fifth, protocolized rescue bicarbonate—applied by identical criteria in both arms but required more often in the control group—may have attenuated between-group differences in acid–base status and day-4 tubular injury markers, biasing conservatively toward the null and likely underestimating the true advantage of RLS. Finally, although point-of-care glucose levels did not differ between groups, the theoretical risk of dextrose-induced tubular injury in the active control arm cannot be entirely excluded.^34^

In conclusion, RLS is superior to a chloride-reduced NSS + D5W regimen in reducing postoperative metabolic acidosis. The physiological benefits extend beyond chloride restriction, and were associated with a higher 1-month eGFR and exploratory signals of reduced early markers of tubular injury and inflammation in ischemia-burdened allografts. Given its clinical efficacy, safety, and economic viability, RLS should be considered a standard perioperative fluid in KT. Future multicenter trials should further elucidate the immunomodulatory mechanisms of lactate buffers on the ischemia-reperfusion cascade.

## Disclosure

All the authors declared no competing interests.

## Supplementary Material

Supplementary file (PDF) **Figure S1.** Daily volumes of protocol i.v. fluid administered during the early postoperative period. (A) Total daily volume of study fluid administered in the control group (NSS + D5W) compared with the intervention group (RLS) from postoperative day 0 (day of surgery) through day 5. (B) Component breakdown of the specific fluids administered per day, illustrating the separate daily volumes of NSS and D5W. Data are presented as mean values; error bars represent 95% CI. **Figure S2.** Longitudinal changes in serum electrolyte concentrations among deceased donor kidney transplant (DDKT) recipients. Postoperative trajectories of (A) serum sodium, (B) serum potassium, (C) serum chloride, and (D) serum bicarbonate from day 0 (baseline) to postoperative day 4. Asterisks indicate a statistically significant difference (*P* < 0.05) between the two groups at that specific time point. Data are presented as mean values; error bars represent 95% CI. **Figure S3:** Longitudinal changes in serum electrolyte concentrations among living donor kidney transplant (LDKT) recipients. Postoperative trajectories of (A) serum sodium, (B) serum potassium, (C) serum chloride, and (D) serum bicarbonate from day 0 (baseline) to postoperative day 4. Asterisks indicate a statistically significant difference (*P* < 0.05) between the two groups at that specific time point. Data are presented as mean values; error bars represent 95% CI. **Figure S4.** Longitudinal changes in kidney allograft function during the first postoperative week among deceased donor kidney transplant (DDKT) recipients. Postoperative trajectories of (A) serum creatinine and (B) estimated glomerular filtration rate (eGFR) from day 0 (baseline) to postoperative day 7. Asterisks indicate a statistically significant difference (*P* < 0.05) between the two groups at that specific time point. Data are presented as mean values; error bars represent 95% CI. **Figure S5.** Longitudinal changes in kidney allograft function during the first postoperative week among living donor kidney transplant (LDKT) recipients. Postoperative trajectories of (A) serum creatinine and (B) estimated glomerular filtration rate (eGFR) from day 0 (baseline) to postoperative day 7. Asterisks indicate a statistically significant difference (p < 0.05) between the two groups at that specific time point. Data are presented as mean values; error bars represent 95% CI. **Figure S6.** Serum inflammatory cytokine concentrations on postoperative day 4 stratified by donor type. Box plots illustrating the distribution of (A) Interferon-gamma (IFN-γ), (B) Tumor Necrosis Factor-alpha (TNF-α), (C) Interleukin-6 (IL-6), and (D) Interleukin-10 (IL-10) levels in the control (NSS + D5W) and intervention (RLS) groups among DDKT and LDKT kidney transplant recipients. The horizontal line within the box represents the median, the lower and upper bounds of the box indicate the interquartile range, and the whiskers extend to the minimum and maximum observed values. **Figure S7.** Requirement and dosage of rescue bicarbonate therapy for severe postoperative metabolic acidosis. (A) The total number of patients in the control (NSS + D5W) and intervention (RLS) groups who developed severe metabolic acidosis (defined as serum bicarbonate < 15 mEq/L) necessitating protocol-directed rescue therapy. (B) Box plot illustrating the total dosage of oral sodium bicarbonate administered over the first 5 postoperative days among the subset of patients who required rescue therapy. The horizontal line within the box represents the median, the lower and upper bounds of the box indicate the interquartile range, and the whiskers extend to the minimum and maximum observed values. **Figure S8:** Longitudinal changes in capillary blood glucose concentrations during the early postoperative period. Postoperative trajectories of point-of-care testing (POCT) glucose from day 0 (day of surgery) through postoperative day 4. The asterisk indicates a statistically significant difference (*P* < 0.05) between the two groups at that specific time point. Data are presented as mean values; error bars represent 95% CI. **Table S1.** Fluid administration dosing and immunosuppressive medication protocol. **Table S2.** Prespecified protocol for rescue bicarbonate therapy for severe postoperative metabolic acidosis. **Table S3.** Cumulative volumes of protocol-directed and nonprotocol i.v. fluids administered during the study period. **Table S4.** Daily volumes of protocol-directed i.v. fluids administered during the early postoperative period. **Table S5.** Daily serum electrolyte concentrations during the early postoperative period: Overall cohort and subgroup analysis by donor type. **Supplementary Table S6.** Adverse events, rescue therapies, and safety outcomes during the early postoperative period. CONSORT checklist. Full protocol and planned statistical analysis.

## Footnotes

Supplementary file (PDF)
**Figure S1.** Daily volumes of protocol i.v. fluid administered during the early postoperative period. (A) Total daily volume of study fluid administered in the control group (NSS + D5W) compared with the intervention group (RLS) from postoperative day 0 (day of surgery) through day 5. (B) Component breakdown of the specific fluids administered per day, illustrating the separate daily volumes of NSS and D5W. Data are presented as mean values; error bars represent 95% CI. **Figure S2.** Longitudinal changes in serum electrolyte concentrations among deceased donor kidney transplant (DDKT) recipients. Postoperative trajectories of (A) serum sodium, (B) serum potassium, (C) serum chloride, and (D) serum bicarbonate from day 0 (baseline) to postoperative day 4. Asterisks indicate a statistically significant difference (*P* < 0.05) between the two groups at that specific time point. Data are presented as mean values; error bars represent 95% CI. **Figure S3.** Longitudinal changes in serum electrolyte concentrations among living donor kidney transplant (LDKT) recipients. Postoperative trajectories of (A) serum sodium, (B) serum potassium, (C) serum chloride, and (D) serum bicarbonate from day 0 (baseline) to postoperative day 4. Asterisks indicate a statistically significant difference (*P* < 0.05) between the two groups at that specific time point. Data are presented as mean values; error bars represent 95% CI. **Figure S4.** Longitudinal changes in kidney allograft function during the first postoperative week among deceased donor kidney transplant (DDKT) recipients. Postoperative trajectories of (A) serum creatinine and (B) estimated glomerular filtration rate (eGFR) from day 0 (baseline) to postoperative day 7. Asterisks indicate a statistically significant difference (*P* < 0.05) between the two groups at that specific time point. Data are presented as mean values; error bars represent 95% CI. **Figure S5:** Longitudinal changes in kidney allograft function during the first postoperative week among living donor kidney transplant (LDKT) recipients. Postoperative trajectories of (A) serum creatinine and (B) estimated glomerular filtration rate (eGFR) from day 0 (baseline) to postoperative day 7. Asterisks indicate a statistically significant difference (*P* < 0.05) between the two groups at that specific time point. Data are presented as mean values; error bars represent 95% CI. **Figure S6:** Serum inflammatory cytokine concentrations on postoperative day 4 stratified by donor type. Box plots illustrating the distribution of (A) Interferon-gamma (IFN-γ), (B) Tumor Necrosis Factor-alpha (TNF-α), (C) Interleukin-6 (IL-6), and (D) Interleukin-10 (IL-10) levels in the control (NSS + D5W) and intervention (RLS) groups among DDKT and LDKT kidney transplant recipients. The horizontal line within the box represents the median, the lower and upper bounds of the box indicate the interquartile range, and the whiskers extend to the minimum and maximum observed values. **Figure S7:** Requirement and dosage of rescue bicarbonate therapy for severe postoperative metabolic acidosis. (A) The total number of patients in the control (NSS + D5W) and intervention (RLS) groups who developed severe metabolic acidosis (defined as serum bicarbonate < 15 mEq/L) necessitating protocol-directed rescue therapy. (B) Box plot illustrating the total dosage of oral sodium bicarbonate administered over the first 5 postoperative days among the subset of patients who required rescue therapy. The horizontal line within the box represents the median, the lower and upper bounds of the box indicate the interquartile range, and the whiskers extend to the minimum and maximum observed values. **Figure S8:** Longitudinal changes in capillary blood glucose concentrations during the early postoperative period. Postoperative trajectories of point-of-care testing (POCT) glucose from day 0 (day of surgery) through postoperative day 4. The asterisk indicates a statistically significant difference (*P* < 0.05) between the two groups at that specific time point. Data are presented as mean values; error bars represent 95% CI. **Table S1.** Fluid administration dosing and immunosuppressive medication protocol. **Table S2.** Prespecified protocol for rescue bicarbonate therapy for severe postoperative metabolic acidosis. **Table S3.** Cumulative volumes of protocol-directed and nonprotocol i.v. fluids administered during the study period. **Table S4.** Daily volumes of protocol-directed i.v. fluids administered during the early postoperative period. **Table S5.** Daily serum electrolyte concentrations during the early postoperative period: Overall cohort and subgroup analysis by donor type. **Table S6.** Adverse events, rescue therapies, and safety outcomes during the early postoperative period. CONSORT checklist. Full protocol and planned statistical analysis.
