# Supplementary material for: Randomized Clinical Trial of Ringer’s Lactate Versus Chloride-Reduced Saline-Dextrose in Postoperative Kidney Transplantation
Source: Kidney Int Rep. 2026 Sep 17;11(11):107081. doi: 10.1016/j.ekir.2026.107081 (PMC13677585; doi:10.1016/j.ekir.2026.107081)
Supplement: Supplementary file 1 [file mmc1.pdf]

Randomized Clinical Trial of Ringer's Lactate versus Chloride-Reduced Saline-Dextrose in Post-operative Kidney Transplantation

Kornrawee Sawasdichai[1], Chanyanuch Rakpithayanon[1], Sirihatai Konwai[2], Thunyatorn Wuttiputhanun[1,3], Nuttha Lumlertgul[1], Kirada Apisutimaitri[4], Rattanaporn Burimsittichai[4], Athaya Vorasittha[3,5], Pea Pobpan[5], Adis Tasanarong[6], Asada Leelahavanichkul[7,8], Natavudh Townamchai[1,3,9], Kearkiat Praditpornsilpa[1], Somchai Eiam-Ong[1], Yingyos Avihingsanon[1,3,9], Suwasin Udomkarjananun[1,3,9]

[1]Division of Nephrology, Department of Medicine, Faculty of Medicine, Chulalongkorn University and King Chulalongkorn Memorial Hospital, Bangkok, Thailand
[2]Division of Nephrology, Department of Medicine, Faculty of Medicine, Prince of Songkla University, Songkla, Thailand
[3]Excellence Center for Solid Organ Transplantation, King Chulalongkorn Memorial Hospital, Thai Red Cross Society, Bangkok, Thailand
[4]Department of Anesthesiology, Faculty of Medicine, Chulalongkorn University and King Chulalongkorn Memorial Hospital, Bangkok, Thailand
[5]Department of Surgery, Faculty of Medicine, Chulalongkorn University and King Chulalongkorn Memorial Hospital, Bangkok, Thailand
[6]Division of Nephrology, Department of Internal Medicine, Faculty of Medicine, Thammasat University, Pathum Thani, Thailand
[7]Center of Excellence on Translation Research in Inflammation and Immunology (CETRII), Department of Microbiology, Chulalongkorn University, Bangkok, Thailand
[8]Department of Microbiology, Faculty of Medicine, Chulalongkorn University and King Chulalongkorn Memorial Hospital, Bangkok, Thailand
[9]Center of Excellence Renal Immunology and Renal Transplantation, Faculty of Medicine, Chulalongkorn University, Bangkok, Thailand

Running title: RLS versus NSS+D5W for Post-KT Fluid Management

Keywords: fluid management, kidney transplantation, Normal Saline Solution, Ringer's Lactate Solution, peri-operation, post-operation

Corresponding author: Suwasin Udomkarjananun, MD, Ph.D.

Supplementary Table S1: Fluid administration dosing and immunosuppressive medication protocol.

Fluid Titration Algorithm	**Control Group (NSS + D5W):**<br>• When the hourly UO was 200 mL or greater, D5W was infused at a fixed rate of 100 mL/hr, while NSS was administered concurrently at a rate calculated as the total UO from the previous hour minus 100 mL. During periods of lower urine output (UO < 200 mL/hr), such as the intraoperative or early postoperative phases, the infusion rates were adjusted to prevent excessive free-water administration; specifically, the D5W and NSS rates were each titrated to match exactly 50% of the previous hour's total UO<br><br>**Intervention Group (RLS):**<br><br>• RLS was administered as a single continuous infusion, with the hourly rate matched exactly to the total UO of the previous hour, regardless of the output volume.
Immunosuppressive medication	• Induction therapy was individualized based on immunologic risk, utilizing either basiliximab or anti-thymocyte globulin (ATG). Intraoperative methylprednisolone (1,000 mg) was administered, followed by 500 mg daily for two days, before transitioning to oral prednisolone.<br>• Maintenance immunosuppression consisted of tacrolimus (target trough concentrations: 7–12 ng/mL for the first 3 months, then 5–8 ng/mL), mycophenolic acid (equivalent to 1,500 mg/day of mycophenolate mofetil), and prednisolone (initiated at 1 mg/kg/day, tapered to 5 mg/day within 3 months post-transplantation).

Supplementary Table S2: Prespecified protocol for rescue bicarbonate therapy for severe postoperative metabolic acidosis.

**Serum HCO_3^- (mEq/L)**	**Intervention**
14.0 – 14.9	Oral Sodamint® 300 mg, 3 tablets, three times daily
12.0 – 13.9	Oral Sodamint® 300 mg, 4 tablets, three times daily
10.0 – 11.9	- Perform arterial blood gas (ABG), serum ketone, and lactate<br>- Administer 7.5% sodium bicarbonate 50 mL IV infusion over 1 hour<br>- Repeat ABG and serum electrolytes after infusion
< 10.0	- Perform ABG, serum ketone, and lactate<br>- Administer 7.5% sodium bicarbonate 100 mL IV infusion over 1 hour<br>- Repeat ABG and serum electrolytes after infusion

Supplementary Table S3: Cumulative volumes of protocol-directed and non-protocol intravenous fluids administered during the study period.

	**RLS (n=67)**	**NSS + D5W (n=67)**	***P* value**
Total protocol IV fluid, mL, mean (SD)	9,521.57 (4,726.12)	9,944.52 (4,723.03)	.61
DDKT	8,362.50 (3,946.38)	8,079.85 (3,897.02)	.73
LDKT	12,060.48 (5,367.51)	14,029.05 (3,732.04)	.18
Total fluid for medication dilution, mL, mean (SD)	630.92 (551.03)	600.63 (373.59)	.79
NSS	577.86 (436.15)	564.34 (267.45)	.88
D5W	53.06 (223.27)	36.29 (170.19)	.72

Abbreviation: DDKT; deceased donor kidney transplantation, LDKT; living donor kidney transplant, NSS+D5W; normal saline solution plus 5%dextrose water, RLS; Ringer's lactate solution

Supplementary Table S4: Daily volumes of protocol-directed intravenous fluids administered during the early postoperative period.

	RLS (n=67)	NSS + D5W (n=67)	*P* value
Day 0, mL, mean (SD)	2,981.64 (1,701.66)	3,108.28 (1,887.91)	.68
Day 1, mL, mean (SD)	3,778.73 (1,895.95)	3,851.39 (2,118.84)	.83
Day 2, mL, mean (SD)	1,689.48 (1,450.88)	1,918.13 (1,195.23)	.32
Day 3, mL, mean (SD)	685.07 (981.59)	782.24 (888.30)	.55
Day 4, mL, mean (SD)	293.81 (625.02)	233.48 (515.69)	.55

Abbreviation: DDKT; deceased donor kidney transplantation, LDKT; living donor kidney transplant, NSS+D5W; normal saline solution plus 5%dextrose water, RLS; Ringer's lactate solution

Supplementary Table S5: Daily serum electrolyte concentrations during the early postoperative period: Overall cohort and subgroup analysis by donor type.

**Overall**	**RLS (n=67)**	**NSS + D5W (n=67)**	***P* value**
Sodium, mEq/L, mean (SD)			
Day0	137.94 (2.45)	138.55 (2.33)	.14
Day1	136.24 (3.23)	134.45 (2.82)	<.001
Day2	135.84 (3.33)	134.57 (3.20)	.03
Day3	135.42 (3.79)	134.36 (5.16)	.18
Day4	134.90 (4.04)	135.04 (3.42)	.82
Potassium, mEq/L, mean (SD)			
Day0	4.16 (0.56)	4.15 (0.58)	.90
Day1	4.37 (0.59)	4.36 (0.62)	.90
Day2	4.31 (0.47)	4.36 (0.50)	.61
Day3	4.23 (0.48)	4.27 (0.48)	.57
Day4	4.03 (0.47)	4.03 (0.51)	.96
Chloride, mEq/L, mean (SD)			

Day0	100.96 (3.39)	101.61 (3.46)	.27
Day1	101.69 (3.79)	102.34 (4.22)	.34
Day2	103.72 (5.16)	105.84 (5.41)	.02
Day3	105.21 (6.44)	107.60 (6.00)	.03
Day4	105.99 (6.53)	107.97 (6.39)	.08
Bicarbonate, mEq/L, mean (SD)			
Day0	23.52 (3.52)	24.15 (4.03)	0.34
Day1	20.64 (2.91)	19.27 (2.78)	0.006
Day2	20.72 (2.55)	17.45 (2.48)	<0.001
Day3	19.79 (2.42)	16.91 (2.25)	<0.001
Day4	19.39 (2.50)	17.31 (2.15)	<0.001

**DDKT**	**RLS (n=46)**	**NSS + D5W (n=46)**	**p-value**
Sodium, mEq/L, mean (SD)			
Day0	137.98 (2.52)	139.11 (1.90)	.02
Day1	135.09 (2.97)	133.65 (2.54)	.01
Day2	135.11 (3.55)	134.19 (3.36)	.20

Day3	134.67 (4.07)	134.63 (3.81)	.96
Day4	134.28 (4.31)	134.72 (3.62)	.60
Potassium, mEq/L, mean (SD)			
Day0	4.22 (0.59)	4.21 (0.61)	.92
Day1	4.52 (0.62)	4.52 (0.62)	.97
Day2	4.33 (0.50)	4.42 (0.52)	.44
Day3	4.17 (0.47)	4.22 (0.49)	.59
Day4	4.00 (0.49)	3.90 (0.48)	.34
Chloride, mEq/L, mean (SD)			
Day0	100.11 (2.73)	100.74 (3.27)	.32
Day1	100.59 (3.16)	101.09 (3.55)	.48
Day2	101.57 (4.39)	103.96 (4.51)	.01
Day3	102.76 (5.98)	105.80 (5.76)	.01
Day4	103.91 (6.64)	106.24 (6.40)	.09
Bicarbonate, mEq/L, mean (SD)			
Day0	23.80 (3.40)	25.09 (3.62)	0.08

Day1	20.00 (2.79)	18.83 (2.89)	0.051
Day2	20.96 (2.62)	17.50 (2.58)	< 0.001
Day3	20.24 (2.50)	16.96 (2.25)	< 0.001
Day4	19.83 (2.56)	17.30 (2.15)	< 0.001
**LDKT**	**RLS (n=21)**	**NSS + D5W (n=21)**	**p-value**
Sodium, mEq/L, mean (SD)			
Day0	137.86 (2.37)	137.33 (2.74)	.51
Day1	138.76 (2.21)	136.19 (2.68)	.002
Day2	137.43 (2.09)	135.38 (2.71)	.009
Day3	137.05 (2.46)	135.48 (3.03)	.07
Day4	136.24 (3.05)	135.76 (2.90)	.61
Potassium, mEq/L, mean (SD)			
Day0	4.03 (0.47)	4.02 (0.50)	.95
Day1	4.04 (0.37)	4.01 (0.47)	.80
Day2	4.28 (0.40)	4.23 (0.45)	.75
Day3	4.35 (0.50)	4.39 (0.44)	.82

Day4	4.09 (0.45)	4.29 (0.46)	.16
Chloride, mEq/L, mean (SD)			
Day0	102.81 (3.98)	103.52 (3.14)	.52
Day1	104.09 (3.99)	105.10 (4.33)	.44
Day2	108.43 (3.26)	109.95 (4.98)	.25
Day3	110.57 (3.49)	111.52 (4.53)	.45
Day4	110.52 (3.12)	111.76 (4.55)	.31
Bicarbonate, mEq/L, mean (SD)			
Day0	22.90 (3.78)	22.10 (4.22)	0.52
Day1	22.05 (2.73)	20.24 (2.30)	0.025
Day2	20.19 (2.34)	17.33 (2.29)	< 0.001
Day3	18.81 (1.94)	16.81 (2.32)	0.004
Day4	18.43 (2.11)	17.33 (2.20)	0.11

Abbreviation: DDKT; deceased donor kidney transplantation, LDKT; living donor kidney transplant, NSS+D5W; normal saline solution plus 5%dextrose water, RLS; Ringer's lactate solution

Supplementary Table S6: Adverse events, rescue therapies, and safety outcomes during the early postoperative period.

	RLS (n=67)	NSS + D5W (n=67)	*P* value
Needed rescue treatment for metabolic acidosis, n (%)	12 (17.9)	39 (58.2)	<.001
Dosage of oral sodium bicarbonate in recipients who needed rescue treatment, tabs in total (day 0-4), mean (SD)	15.08 (11.98)	19.38 (15.70)	.38
Incidence of hyperkalemia, n (%)	16 (23.9)	18 (26.9)	.69
Dosage of Sodium Polystyrene Sulfonate, grams in total (day 0-4), mean (SD)	16.19 (13.35)	13.83 (9.76)	.56
POCT glucose, mg/dL, mean (SD)			
Day 0	156.74 (35.54)	169.94 (49.63)	.08
Day 1	157.49 (42.28)	172.00 (34.94)	.032
Day 2	166.14 (47.00)	154.87 (26.66)	.09
Day 3	153.74 (40.00)	149.00 (32.93)	.46
Day 4	133.76 (35.93)	131.24 (38.79)	.69
POCT glucose > 180 mg/dL, n (%)	11 (16.4)	15 (22.4)	.76
Hemodynamic instability, n (%)	3 (4.5)	2 (3.0)	.65

Abbreviation: DDKT; deceased donor kidney transplantation, LDKT; living donor kidney transplant, NSS+D5W; normal saline solution plus 5%dextrose water, POCT; point of care testing, RLS; Ringer’s lactate solution

Supplementary Figure S1: Daily volumes of protocol intravenous fluid administered during the early postoperative period. (A) Total daily volume of study fluid administered in the control group (NSS + D5W) compared to the intervention group (RLS) from postoperative day 0 (day of surgery) through day 5. (B) Component breakdown of the specific fluids administered per day, illustrating the separate daily volumes of NSS and D5W. Data are presented as mean values; error bars represent 95% CI.

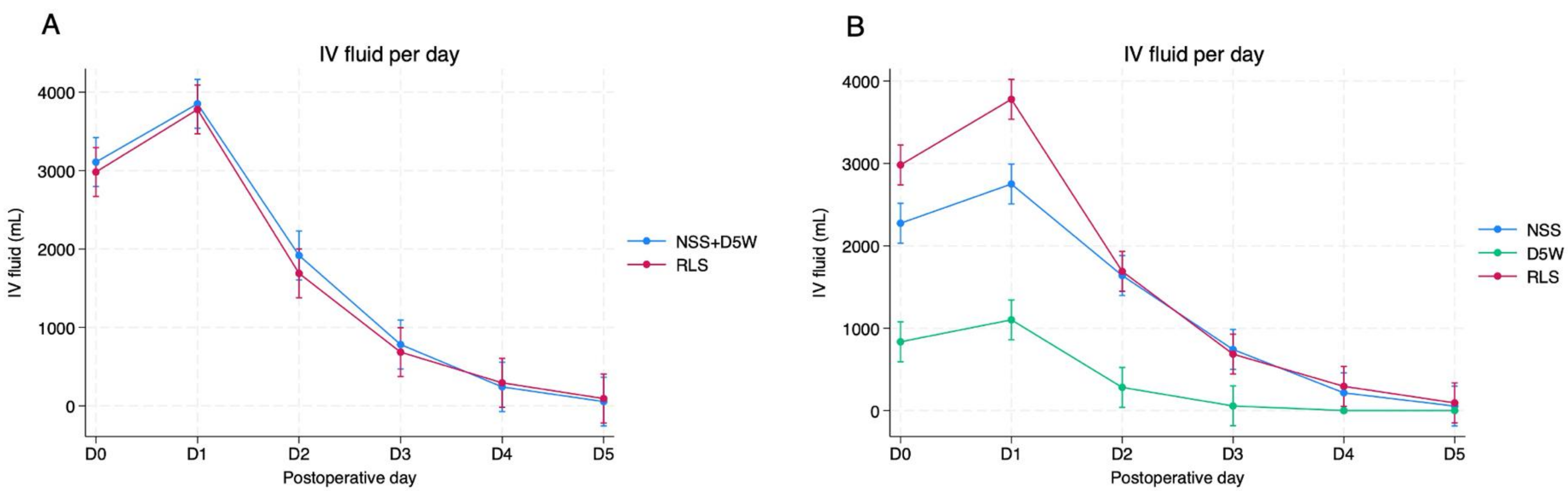

Supplementary Figure S2: Longitudinal changes in serum electrolyte concentrations among deceased donor kidney transplant (DDKT) recipients. Postoperative trajectories of (A) serum sodium, (B) serum potassium, (C) serum chloride, and (D) serum bicarbonate from day 0 (baseline) to postoperative day 4. Asterisks indicate a statistically significant difference ($p < 0.05$) between the two groups at that specific time point. Data are presented as mean values; error bars represent 95% CI.

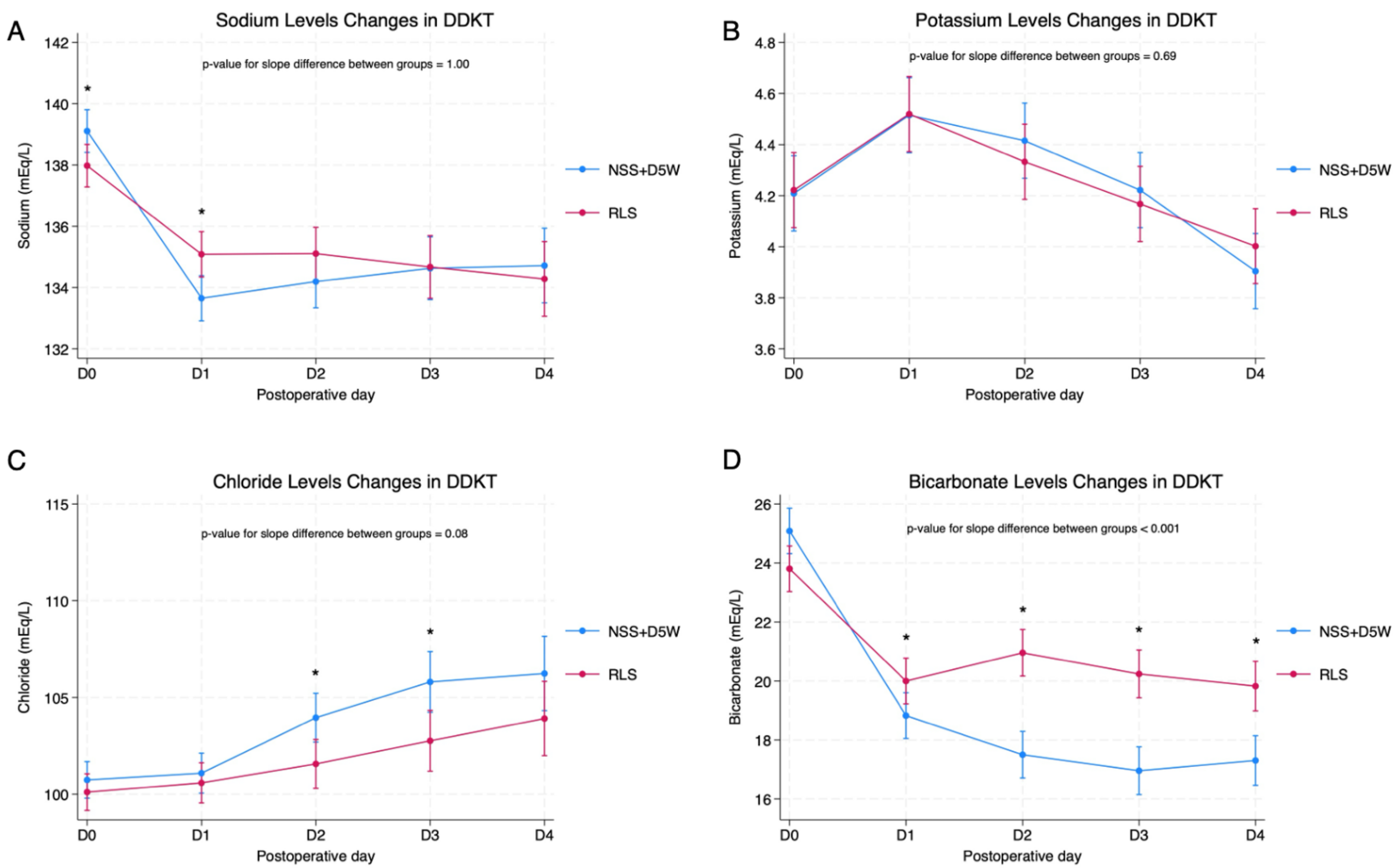

Supplementary Figure S3: Longitudinal changes in serum electrolyte concentrations among living donor kidney transplant (LDKT) recipients. Postoperative trajectories of (A) serum sodium, (B) serum potassium, (C) serum chloride, and (D) serum bicarbonate from day 0 (baseline) to postoperative day 4. Asterisks indicate a statistically significant difference ($p < 0.05$) between the two groups at that specific time point. Data are presented as mean values; error bars represent 95% CI.

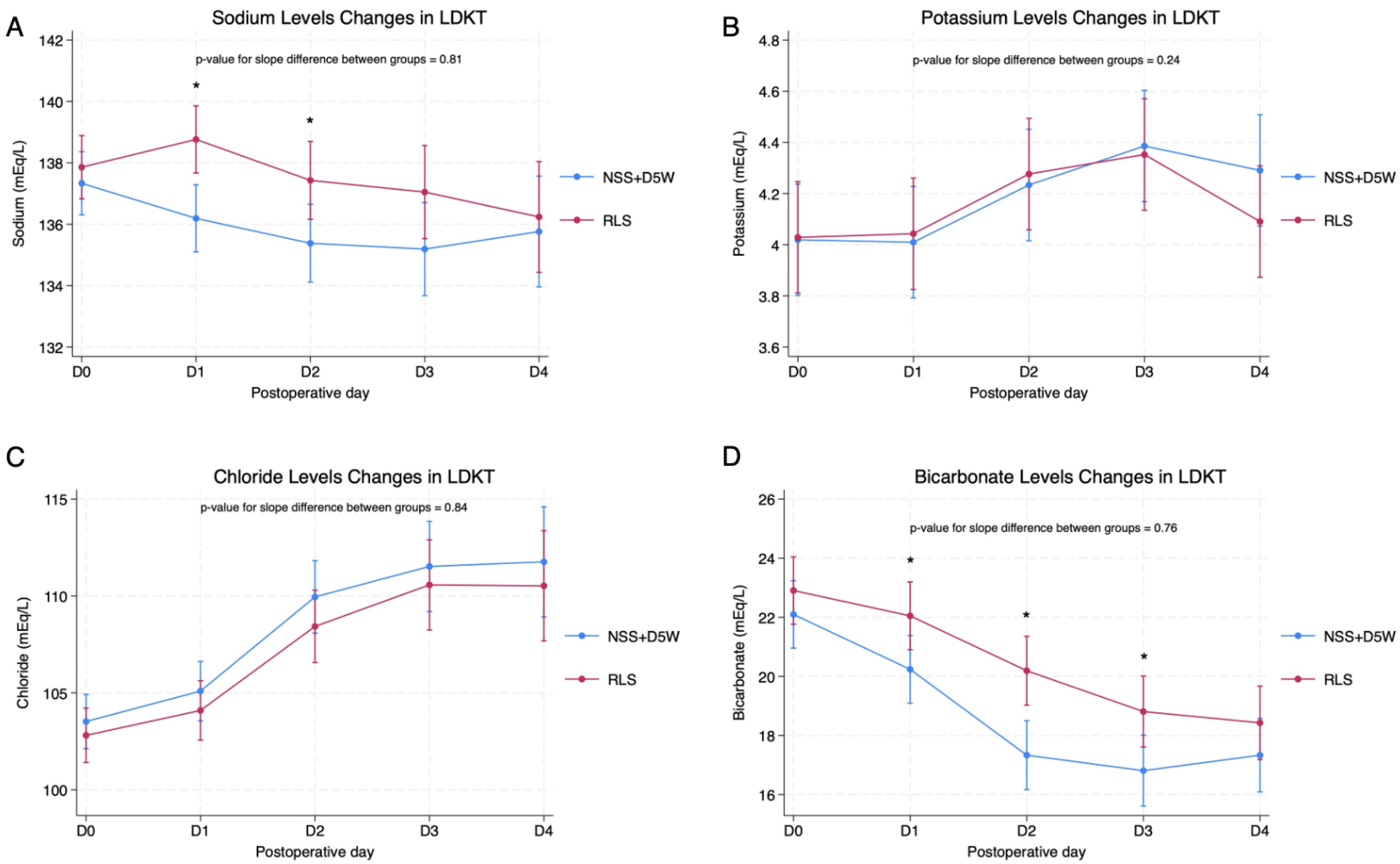

Supplementary Figure S4: Longitudinal changes in kidney allograft function during the first postoperative week among deceased donor kidney transplant (DDKT) recipients. Postoperative trajectories of (A) serum creatinine and (B) estimated glomerular filtration rate (eGFR) from day 0 (baseline) to postoperative day 7. Asterisks indicate a statistically significant difference ($p < 0.05$) between the two groups at that specific time point. Data are presented as mean values; error bars represent 95% CI.

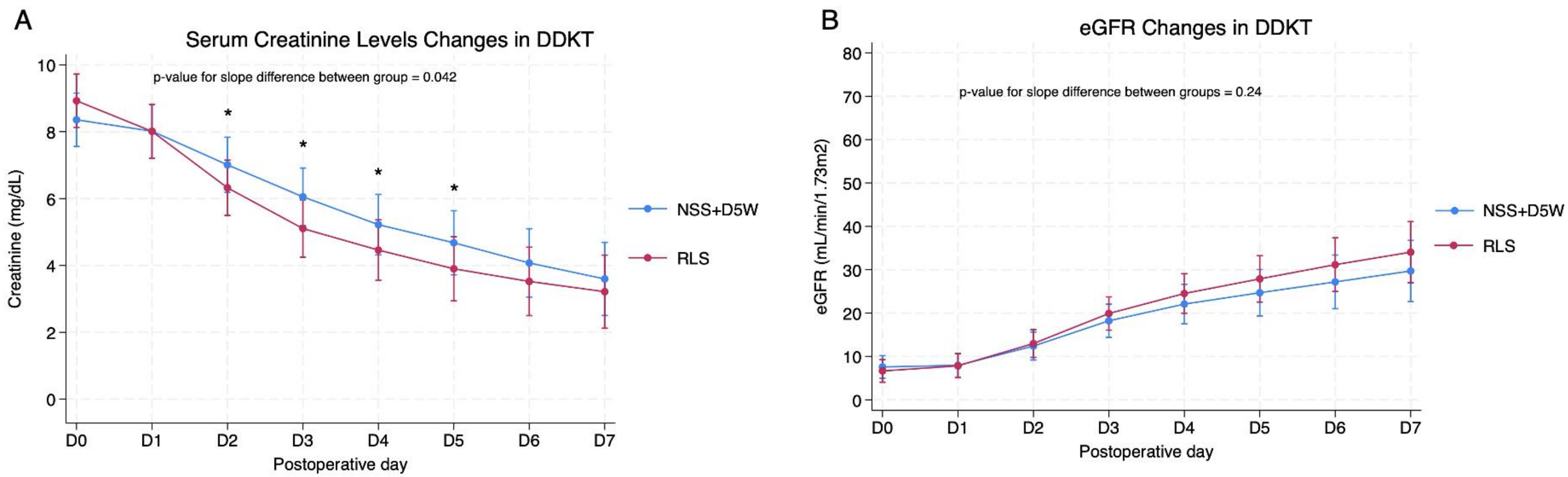

Supplementary Figure S5: Longitudinal changes in kidney allograft function during the first postoperative week among living donor kidney transplant (LDKT) recipients. Postoperative trajectories of (A) serum creatinine and (B) estimated glomerular filtration rate (eGFR) from day 0 (baseline) to postoperative day 7. Asterisks indicate a statistically significant difference ($p < 0.05$) between the two groups at that specific time point. Data are presented as mean values; error bars represent 95% CI.

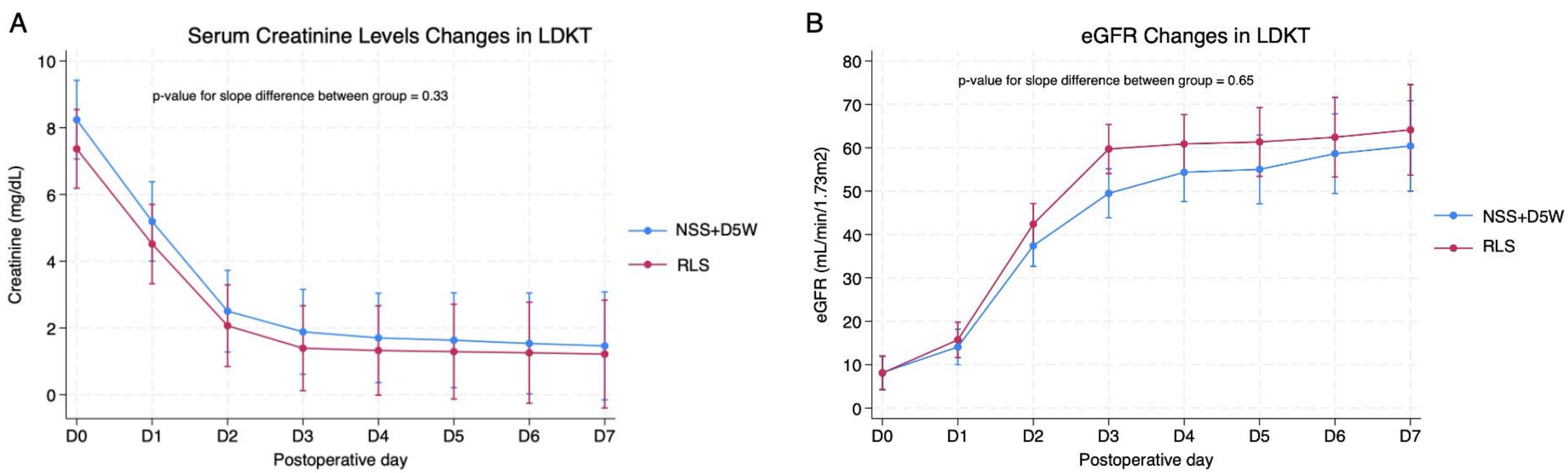

Supplementary Figure S6: Serum inflammatory cytokine concentrations on postoperative day 4 stratified by donor type. Box plots illustrating the distribution of (A) Interferon-gamma (IFN-γ), (B) Tumor Necrosis Factor-alpha (TNF-α), (C) Interleukin-6 (IL-6), and (D) Interleukin-10 (IL-10) levels in the control (NSS + D5W) and intervention (RLS) groups among DDKT and LDKT kidney transplant recipients. The horizontal line within the box represents the median, the lower and upper bounds of the box indicate the interquartile range, and the whiskers extend to the minimum and maximum observed values.

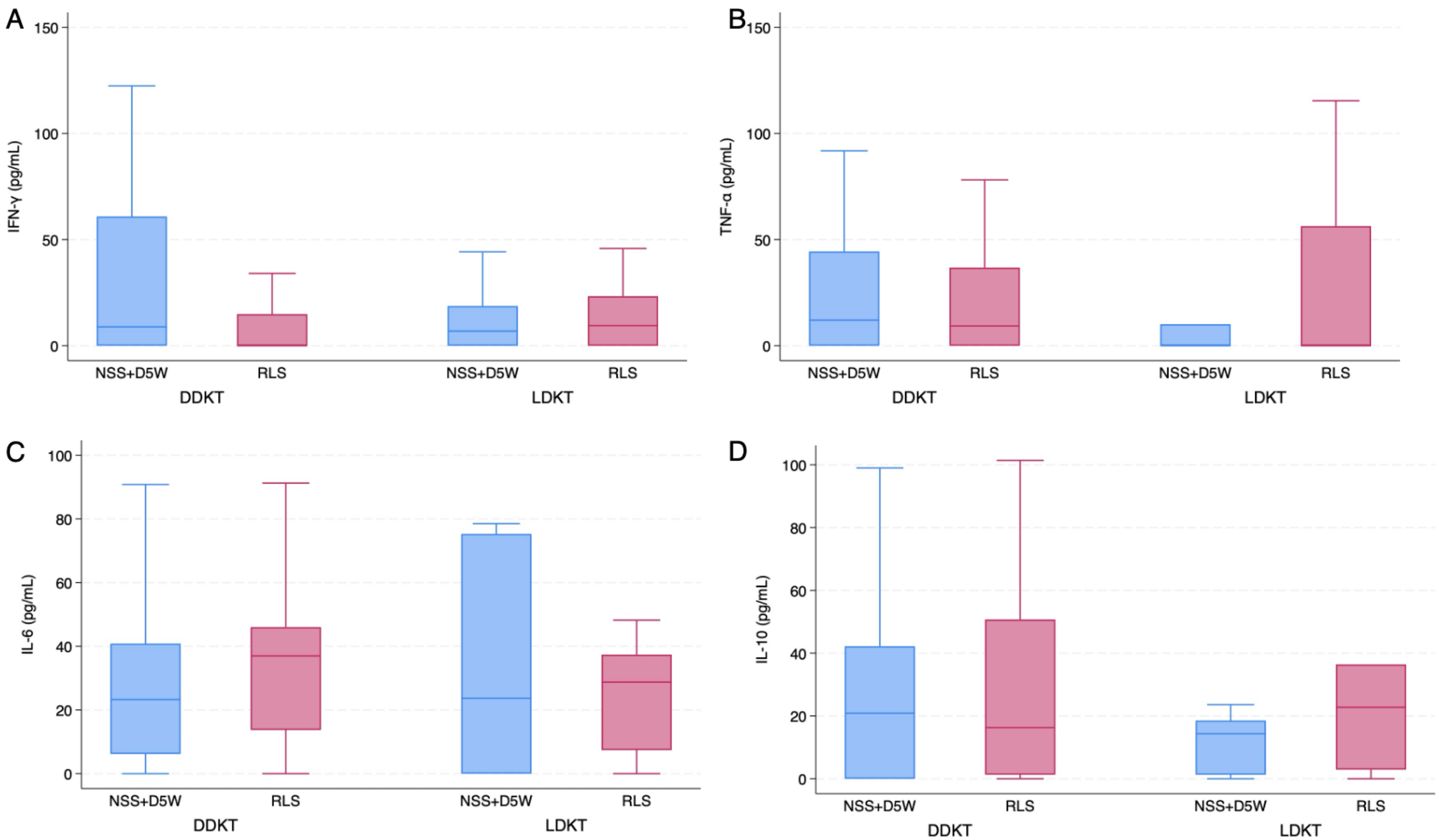

Supplementary Figure S7: Requirement and dosage of rescue bicarbonate therapy for severe postoperative metabolic acidosis. (A) The total number of patients in the control (NSS + D5W) and intervention (RLS) groups who developed severe metabolic acidosis (defined as serum bicarbonate < 15 mEq/L) necessitating protocol-directed rescue therapy. (B) Box plot illustrating the total dosage of oral sodium bicarbonate administered over the first 5 postoperative days among the subset of patients who required rescue therapy. The horizontal line within the box represents the median, the lower and upper bounds of the box indicate the interquartile range, and the whiskers extend to the minimum and maximum observed values.

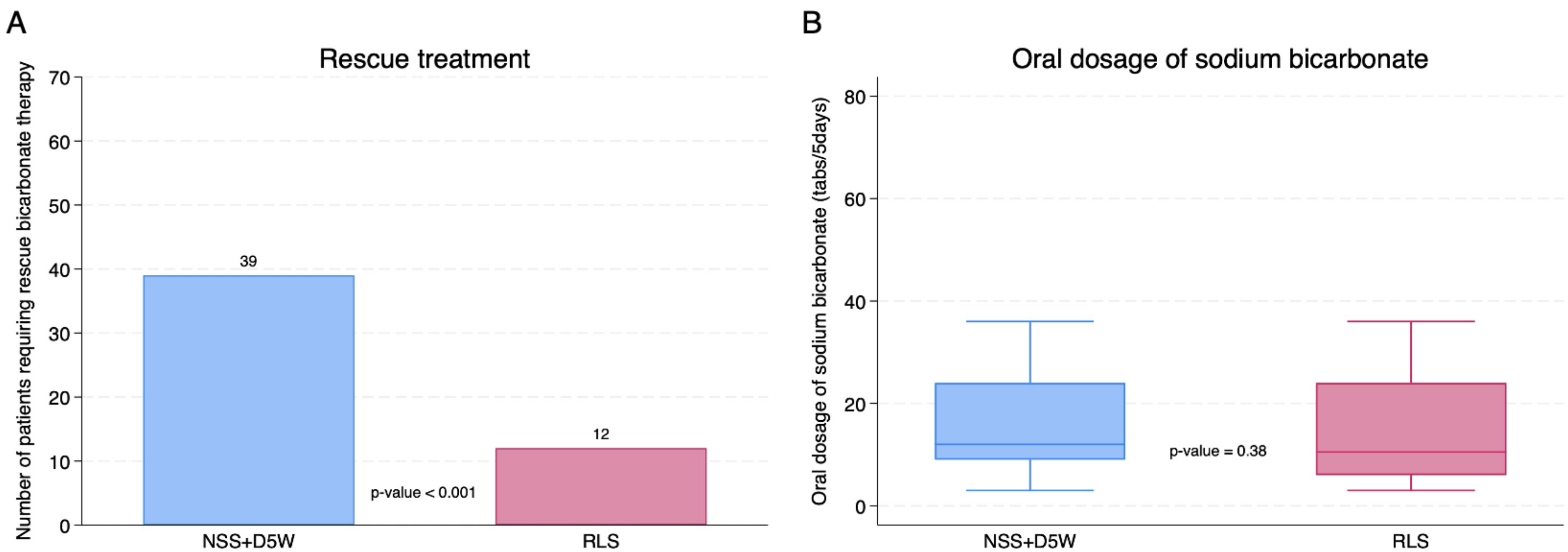

Supplementary Figure S8: Longitudinal changes in capillary blood glucose concentrations during the early postoperative period. Postoperative trajectories of point-of-care testing (POCT) glucose from day 0 (day of surgery) through postoperative day 4. The asterisk indicates a statistically significant difference ($p < 0.05$) between the two groups at that specific time point. Data are presented as mean values; error bars represent 95% CI.

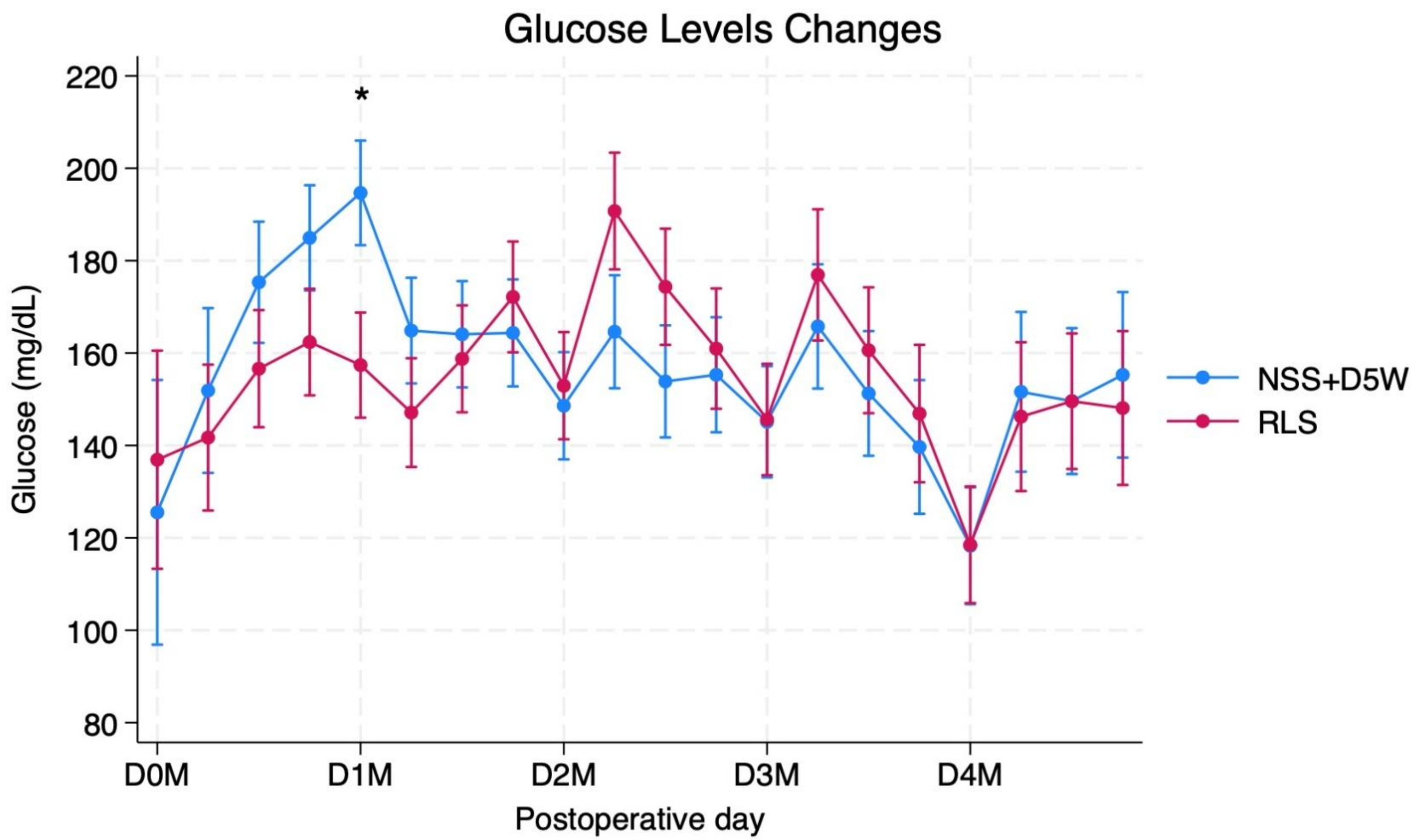

CONSORT 2025 checklist of information to include when reporting a randomised trial*

Section / Topic	No	CONSORT 2025 checklist item description	Reported on page no.
**Title and abstract**			
Title and structured abstract	1a	Identification as a randomised trial	p. 1
	1b	Structured summary of the trial design, methods, results, and conclusions	p. 3-4
**Open science**			
Trial registration	2	Name of trial registry, identifying number (with URL) and date of registration	p. 4, 6
Protocol and statistical analysis plan	3	Where the trial protocol and statistical analysis plan can be accessed	p. 24 (Supplementary File: full protocol and planned statistical analysis)
Data sharing	4	Where and how the individual de-identified participant data (including data dictionary), statistical code and any other materials can be accessed	p. 22
Funding and conflicts of interest	5a	Sources of funding and other support (e.g., supply of drugs), and role of funders in the design, conduct, analysis and reporting of the trial	p. 22
	5b	Financial and other conflicts of interest of the manuscript authors	p. 21
**Introduction**			
Background and rationale	6	Scientific background and rationale	p. 5
Objectives	7	Specific objectives related to benefits and harms	p. 5
**Methods**			
Patient and public involvement	8	Details of patient or public involvement in the design, conduct and reporting of the trial	p. 21
Trial design	9	Description of trial design including type of trial (e.g., parallel group, crossover), allocation ratio, and framework (e.g., superiority, equivalence, non-inferiority, exploratory)	p. 6
Changes to trial protocol	10	Important changes to the trial after it commenced including any outcomes or analyses that were not prespecified, with reason	Not applicable

Trial setting	11	Settings (e.g., community, hospital) and locations (e.g., countries, sites) where the trial was conducted	p. 6
Eligibility criteria	12a	Eligibility criteria for participants	p. 6
	12b	If applicable, eligibility criteria for sites and for individuals delivering the interventions (e.g., surgeons, physiotherapists)	Not applicable
Intervention and comparator	13	Intervention and comparator with sufficient details to allow replication. If relevant, where additional materials describing the intervention and comparator (e.g., intervention manual) can be accessed	p. 6
Outcomes	14	Pre-specified primary and secondary outcomes, including the specific measurement variable (e.g., systolic blood pressure), analysis metric (e.g., change from baseline, final value, time to event), method of aggregation (e.g., median, proportion), and time point for each outcome	p. 7-8
Harms	15	How harms were defined and assessed (e.g., systematically, non-systematically)	p. 8, 13
Sample size	16a	How sample size was determined, including all assumptions supporting the sample size calculation	p. 9
	16b	Explanation of any interim analyses and stopping guidelines	Not applicable
Randomisation:			
Sequence generation	17a	Who generated the random allocation sequence and the method used	p. 6
	17b	Type of randomisation and details of any restriction (e.g., stratification, blocking and block size)	p. 6
Allocation concealment mechanism	18	Mechanism used to implement the random allocation sequence (e.g., central computer/telephone; sequentially numbered, opaque, sealed containers), describing any steps to conceal the sequence until interventions were assigned	p. 6
Implementation	19	Whether the personnel who enrolled and those who assigned participants to the interventions had access to the random allocation sequence	p. 6
Blinding	20a	Who was blinded after assignment to interventions (e.g., participants, care providers, outcome assessors, data analysts)	p. 6
	20b	If blinded, how blinding was achieved and description of the similarity of interventions	p. 6 (open-label; blinding not applicable)
Statistical methods	21a	Statistical methods used to compare groups for primary and secondary outcomes, including harms	p. 9-10
	21b	Definition of who is included in each analysis (e.g., all randomised participants), and in which group	p. 9-10
	21c	How missing data were handled in the analysis	p. 9-10

	21d	Methods for any additional analyses (e.g., subgroup and sensitivity analyses), distinguishing prespecified from post-hoc	p. 9-10
**Results**			
Participant flow, including flow diagram	22a	For each group, the numbers of participants who were randomly assigned, received intended intervention, and were analysed for the primary outcome	p. 10; Figure 1 (legend p. 27)
	22b	For each group, losses and exclusions after randomisation, together with reasons	p. 10; Figure 1 (legend p. 27)
Recruitment	23a	Dates defining the periods of recruitment and follow-up for outcomes of benefits and harms	p. 10
	23b	If relevant, why the trial ended or was stopped	Not applicable
Intervention and comparator delivery	24a	Intervention and comparator as they were actually administered (e.g., where appropriate, who delivered the intervention/comparator, how participants adhered, whether they were delivered as intended [fidelity])	p. 6, 10-11
	24b	Concomitant care received during the trial for each group	p. 6-7
Baseline data	25	A table showing baseline demographic and clinical characteristics for each group	p. 28-29 (Table 1)
Numbers analysed, outcomes and estimation	26	For each primary and secondary outcome, by group:<br>● the number of participants included in the analysis<br>● the number of participants with available data at the outcome time point<br>● result for each group, and the estimated effect size and its precision (such as 95% confidence interval)<br>● for binary outcomes, presentation of both absolute and relative effect size	p. 10-13, 30-31, 33 (Tables 2-3)
Harms	27	All harms or unintended events in each group	p. 13
Ancillary analyses	28	Any other analyses performed, including subgroup and sensitivity analyses, distinguishing pre-specified from post-hoc	p. 11-13
**Discussion**			
Interpretation	29	Interpretation consistent with results, balancing benefits and harms, and considering other relevant evidence	p. 14-20
Limitations	30	Trial limitations, addressing sources of potential bias, imprecision, generalisability, and, if relevant, multiplicity of analyses	p. 18-19

*We strongly recommend reading this statement in conjunction with the CONSORT 2025 Explanation and Elaboration and/or the CONSORT 2025 Expanded Checklist for important clarifications on all the items. We also recommend reading relevant CONSORT extensions. See www.consort-spirit.org.

Citation: Hopewell S, Chan AW, Collins GS, Hróbjartsson A, Moher D, Schulz KF, et al. CONSORT 2025 Statement: updated guideline for reporting randomised trials. BMJ. 2025; 388:e081123. https://dx.doi.org/10.1136/bmj-2024-081123.

© 2025 Hopewell et al. This is an Open Access article distributed under the terms of the Creative Commons Attribution License (https://creativecommons.org/licenses/by/4.0/), which permits unrestricted use, distribution, and reproduction in any medium, provided the original work is properly cited.

Research Protocol

IRB No.0281/67

1. Proposal Title

A Pragmatic, Open-Label, Randomized Controlled Trial of Ringer's Lactate Solution Versus Normal Saline Plus 5% Dextrose Solution on Metabolic Acidosis During the First Week of the Post-operative Period After Kidney Transplantation

2. Investigators

Principal Investigator: Kornrawee Sawasdichai, M.D.

Affiliation: Division of Nephrology, Department of Medicine, Faculty of Medicine, Chulalongkorn University

Supervisor: Assistant Professor Suwasin Udomkarnjananun, M.D., Ph.D.

Affiliation: Division of Nephrology, Department of Medicine, Faculty of Medicine, Chulalongkorn University

Protocol Version and Amendment Tracking

Version Number/ Affected Section(s)	Summary of Revisions Made	Protocol Launch Date
Version 1.0	- First draft	-
Version 2.0	- Corrected typographical and grammatical errors.<br>- Revised the Thai title to better align with the English title.	29/3/2024
Version 3.0	- Added the conceptual framework.<br>- Added the exclusion criteria for multi-organ transplantation.<br>- Added details regarding stratified randomization.<br>- Updated the study duration.	6/7/2024
Version 4.0	- Recalculated and increased the sample size to compensate for attrition rate.	13/1/2026

3. Rationale

Chronic kidney disease (CKD) is a common condition and a major public health problem in Thailand. It is a chronic, incurable disease that requires continuous treatment and incurs very high treatment costs, particularly when the disease progresses to end-stage renal disease (ESRD), which necessitates renal replacement therapy (RRT) such as hemodialysis (HD), peritoneal dialysis (PD), or kidney transplantation (KT).[1]

KT is the optimal modality of renal replacement therapy, as it reduces mortality compared with HD and PD.[2] However, kidney transplantation is limited by the number of available donors; therefore, strategies to maximize allograft longevity are of considerable importance. A proportion of patients develop delayed graft function (DGF), defined as the need for dialysis within the first seven days after kidney transplantation.[3] The principal pathophysiological mechanism of DGF is hypothesized to involve ischemia–reperfusion injury (IRI),[4] which induces renal inflammation, activates the immune response, and subsequently triggers allograft rejection, thereby reducing graft survival.[5] In 2009, Yarlagadda et al.[6] performed a meta-analysis of 11 studies involving 4,995 kidney transplant recipients and demonstrated that patients who developed DGF had a significantly higher risk of allograft rejection compared with those who did not. The risk factors for DGF[5] include both non-modifiable donor- and recipient-related factors and modifiable factors such as surgical parameters, ischemic time, early immunosuppressive drug levels, intraoperative and postoperative intravenous fluids, and hemodynamic balance.

One modifiable factor is the choice of intravenous (IV) fluid. At present, the crystalloid solution most commonly used in patients with CKD is 0.9% normal saline (NSS), which has been in widespread clinical use for many decades and was the first crystalloid adopted across virtually all patient populations.[7] The current standard of care for post-kidney-transplant fluid management at King Chulalongkorn Memorial Hospital, and in Thailand generally, consists of hourly urine-matched NSS during the first three to five postoperative days, combined with 5% dextrose in water (5%DW) infused at a fixed rate of 100 mL/hour. This combination is employed to avoid excessive polyuria resulting from salt diuresis, which may delay graft recovery, and to limit the chloride load delivered by normal saline, which can induce hyperchloremia and metabolic acidosis. Metabolic acidosis, in turn, promotes renal vasoconstriction and decreases renal perfusion, contributing to the development of delayed graft function (DGF).[8–9,16]

Nevertheless, owing to continued advances in clinical fluid therapy, there is now growing evidence that balanced crystalloid solutions, such as Ringer's lactate, are more effective than normal saline in preventing acute kidney injury. This benefit is mediated by improved renal blood flow and a lower incidence of metabolic acidosis.[10–12] These observations provide the rationale for the present study, which aims to compare Ringer's lactate solution with normal saline in the first postoperative week after kidney transplantation at King Chulalongkorn Memorial Hospital, with respect to metabolic acidosis and graft function. The findings are intended to inform improvements in post-transplant care. Moreover, because the current protocol at King Chulalongkorn Memorial Hospital

requires concurrent administration of two fluids (normal saline and 5% dextrose), demonstrating superiority of Ringer's lactate would support a transition from a two-fluid regimen to a single-fluid regimen. This change would reduce resource utilization and nursing workload, and it may inform future studies examining delayed graft function.

4. Literature Review

In 2005, a study conducted by anesthesiologists[13] compared intraoperative administration of Ringer's lactate solution with normal saline during kidney transplantation. The study assessed graft function using serum creatinine on postoperative day 3 and evaluated adverse effects between the two groups. The results demonstrated no significant difference in serum creatinine between groups; however, patients who received normal saline had significantly higher rates of hyperkalemia and metabolic acidosis compared with those who received Ringer's lactate solution.

Subsequently, Wan et al.[14] performed a systematic review of six randomized controlled trials (RCTs) comparing intraoperative normal saline with lower-chloride solutions, including Ringer's lactate, Plasma-Lyte, and acetate-containing solutions, during kidney transplantation. No significant difference was observed in the incidence of DGF or hyperkalemia between the groups; however, lower-chloride solutions were associated with a significantly lower incidence of metabolic acidosis compared with normal saline.

In 2021, Saini et al.[15] conducted a three-arm comparative study of normal saline, Ringer's lactate solution, and Plasma-Lyte administered intraoperatively during kidney transplantation, assessing metabolic acidosis at the end of surgery. The normal saline group had statistically greater metabolic acidosis than the other two groups, although the difference was not considered clinically significant (pH 7.29, 7.33, and 7.31, respectively; $p = 0.03$).

More recently, in 2023, Collins et al.[16] performed a large multicenter RCT across 16 centers in Australia and New Zealand, comparing Plasma-Lyte with normal saline in deceased donor kidney transplant recipients. Fluids were administered intraoperatively and for 48 hours postoperatively, with DGF as the primary outcome. Plasma-Lyte was associated with a significantly lower incidence of DGF compared with normal saline, and the investigators recommended that Plasma-Lyte be used as the first-line fluid in deceased donor kidney transplantation.

Regarding the association between normal saline and metabolic acidosis, Etezadi et al.[17] compared tight intraoperative correction of metabolic acidosis (maintaining serum bicarbonate between 20 and 25 mEq/L) with correction only at the onset of severe acidosis (pH < 7.1). Tight acid–base control was associated with superior postoperative renal function compared with correction limited to severe acidosis.

Additionally, Tejchman et al.[18] evaluated serial arterial blood gas measurements during kidney transplantation and demonstrated that, following reperfusion of the allograft, blood acidity increased significantly, a finding that has been proposed as a biomarker of IRI.

The pathophysiology of allograft injury due to IRI begins with renal hypoxia, the severity of which depends on the ischemic time.[19] Hypoxia leads to a reduction in intracellular adenosine triphosphate (ATP), a shift to anaerobic metabolism, increased lactic acid production, intracellular acidosis, and cellular swelling. Upon reperfusion, reactive oxygen species (ROS) and damage-associated molecular patterns (DAMPs) are released, activating innate immunity and inducing leukocytes to secrete pro-inflammatory cytokines, including tumor necrosis factor-alpha (TNF-α), interleukin-1 (IL-1), interleukin-6 (IL-6), interferon-gamma (IFN-γ), interleukin-12 (IL-12), interleukin-10 (IL-10), and interleukin-8 (IL-8). These mediators cause allograft inflammation, activation of adaptive immunity, and subsequent graft rejection.[20]

Serum creatinine is not an ideal biomarker for early acute kidney injury (AKI) because it rises 24–48 hours after the injury and is confounded by factors such as muscle mass and drugs that affect creatinine excretion. Several biomarkers have been proposed as more sensitive indicators of AKI than serum creatinine,[21] including serum neutrophil gelatinase-associated lipocalin (NGAL), urine NGAL, urinary interleukin-18 (IL-18), and kidney injury molecule-1 (KIM-1). Urine NGAL and interleukin-6 (IL-6) have also been identified as prognostic biomarkers following AKI. Studies of cytokine levels that correlate with AKI[22] have shown that serum IL-8, IL-16, IL-18, and TNF, along with urinary IL-8, serve as early indicators of renal injury. IL-8, IL-16, IL-18, and TNF are secreted from inflamed or injured proximal tubular epithelial cells; IL-18 activates leukocytes to secrete IFN-γ, driving innate immunity and amplifying inflammation. Elevated serum levels of IFN-γ, IL-2, IL-8, IL-10, IL-6, transforming growth factor-beta (TGF-β), and IL-18 have also been reported in patients with ischemic AKI.

Kidney transplantation unavoidably involves some degree of renal injury through IRI. Serum creatinine in the early post-transplant period may therefore be unreliable because of the factors noted above and because recipients begin with heterogeneous baseline creatinine values from prior renal replacement therapy. Existing data demonstrate that cytokine levels can detect renal injury early, which motivates investigation of whether different postoperative intravenous fluids may indirectly influence the degree of graft injury.

In summary, the existing literature on fluid therapy in kidney transplantation has focused predominantly on the intraoperative period. No study has yet evaluated fluid management beyond the intraoperative period, apart from the most recent trial extending to 48 hours postoperatively, which was restricted to deceased donor recipients. In clinical practice, however, living donor kidney transplant recipients typically receive a larger fluid volume for a longer duration—often up to 72 hours postoperatively—and are at greater risk of metabolic acidosis. Moreover, the aforementioned study employed Plasma-Lyte, which is costly and not widely available in most Thai hospitals. These considerations provide the rationale for the present study, which aims to

compare Ringer's lactate solution with normal saline in both living donor and deceased donor kidney transplant recipients, with the goal of optimizing postoperative care.

5. Objectives

Primary Objective

- To compare the effect of Ringer's lactate solution with the combined infusion of normal saline and 5% dextrose (the existing standard regimen) on the development of metabolic acidosis during the first week after kidney transplantation, using pH from venous blood gas (VBG) in conjunction with serum bicarbonate (HCO_3^-) to confirm metabolic acidosis.

Secondary Objectives

- To compare the effect of Ringer's lactate solution with normal saline plus 5% dextrose on changes in serum bicarbonate (HCO_3^-) during the first postoperative week after kidney transplantation.
- To compare the effect of Ringer's lactate solution with normal saline plus 5% dextrose on the occurrence of sodium (Na^+) abnormalities during the first postoperative week after kidney transplantation.
- To compare the effect of Ringer's lactate solution with normal saline plus 5% dextrose on the occurrence of hyperkalemia (elevated serum K^+) during the first postoperative week after kidney transplantation.
- To compare the effect of Ringer's lactate solution with normal saline plus 5% dextrose on graft function during the first postoperative week after kidney transplantation.
- To compare the rate of therapeutic interventions required to correct metabolic acidosis between Ringer's lactate solution and normal saline plus 5% dextrose during the first postoperative week after kidney transplantation.
- To compare the incidence of delayed graft function (DGF) between Ringer's lactate solution and normal saline plus 5% dextrose during the first postoperative week after kidney transplantation.
- To compare the effect of Ringer's lactate solution with normal saline plus 5% dextrose on cytokine levels, including TNF-α, IL-6, IFN-γ, and IL-10 during the first postoperative week after kidney transplantation.
- To compare the effect of Ringer's lactate solution with normal saline plus 5% dextrose on urine NGAL during the first postoperative week after kidney transplantation.

6. Research Questions / Hypotheses

Primary Question

- Does Ringer's lactate solution differ from the combined infusion of normal saline and 5% dextrose in its effect on the development of metabolic acidosis during the first week after kidney transplantation?

Secondary Questions

- Does Ringer's lactate solution differ from normal saline plus 5% dextrose in its effect on serum bicarbonate (HCO_3^-) changes during the first week after kidney transplantation?
- Does Ringer's lactate solution differ from normal saline plus 5% dextrose in the occurrence of hyperkalemia during the first week after kidney transplantation?
- Does Ringer's lactate solution differ from normal saline plus 5% dextrose in the occurrence of sodium abnormalities during the first week after kidney transplantation?
- Does Ringer's lactate solution differ from normal saline plus 5% dextrose in its effect on cytokine levels (TNF-α, IL-6, IFN-γ, IL-10) during the first week after kidney transplantation?
- Does Ringer's lactate solution differ from normal saline plus 5% dextrose in its effect on urine NGAL during the first week after kidney transplantation?
- Does Ringer's lactate solution differ from normal saline plus 5% dextrose in the incidence of delayed graft function (DGF) during the first week after kidney transplantation?

7. Conceptual Framework

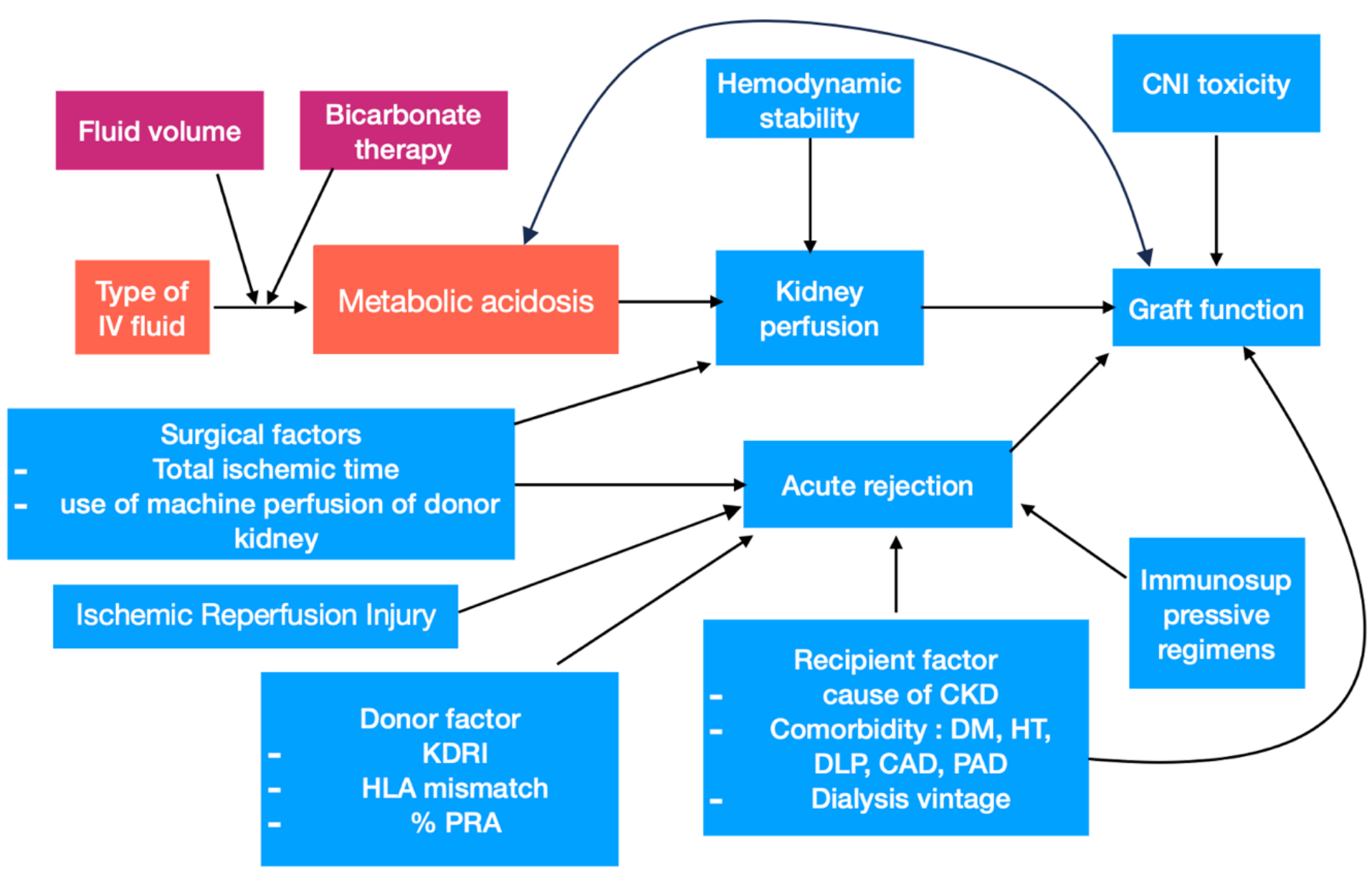

8. Keywords

Balanced crystalloid, Balanced salt solution, Ringer's lactate solution, Crystalloid solutions, Intravenous (IV) fluid, Kidney transplantation, Metabolic acidosis, Delayed graft function (DGF), Graft function, Ischemia–reperfusion injury (IRI), Randomized controlled trial (RCT).

9. Research Design

Open-label, randomized controlled trial (RCT).

10. Research Methodology

Population

Patients undergoing kidney transplantation (KT) at King Chulalongkorn Memorial Hospital.

Approach to Participants

The investigator or the attending physician will invite every eligible patient scheduled for kidney transplantation at King Chulalongkorn Memorial Hospital during the study period to participate. For recipients of living donor kidney transplantation (LDKT), the invitation will occur on the first day of hospital admission, typically two days before surgery. For recipients of deceased donor kidney transplantation (DDKT), the invitation will occur on the day of pre-operative evaluation.

Inclusion Criteria

1. Age ≥ 18 years.

2. Recipients of kidney transplantation at King Chulalongkorn Memorial Hospital from either deceased donors (DDKT) or living donors (LDKT).

Exclusion Criteria

1. Body weight < 20 kg.[16]

2. Recipients of simultaneous multi-organ transplantation.[16,28]

Informed Consent Process

The investigator or the attending physician will explain the background and significance of the study, distribute the participant information sheet and consent form for review, and allow the

patient adequate time for consideration up to the point of entering the operating room. If the patient agrees to participate, written informed consent will be obtained before enrollment.

Study Procedures

1. Patients undergoing kidney transplantation at King Chulalongkorn Memorial Hospital will be randomized into two groups: the Ringer's lactate (LR) group and the normal saline plus 5% dextrose (NSS + 5%DW) group.

2. Preoperative laboratory evaluation will be performed according to standard of care, including complete blood count (CBC), blood urea nitrogen (BUN), serum creatinine (Cr), electrolytes, calcium (Ca), phosphate (P), magnesium (Mg), liver function tests (LFT), prothrombin time (PT), partial thromboplastin time (PTT), and international normalized ratio (INR).

3. Immediately postoperative laboratory evaluation will be performed according to standard of care, including BUN, Cr, electrolytes, and venous blood gas (VBG). VBG pH, interpreted with serum bicarbonate (HCO_3^-), will be used to confirm metabolic (not respiratory) acidosis.

4. After return from the operating room, hourly urine output will be measured. In each group, the IV fluid rate will be titrated hour by hour to match urine output, in accordance with the study protocol and the standard of care.

5. Volume status will be evaluated in all patients via twice-daily weighing (morning and evening), bedside inferior vena cava (IVC) ultrasonography, and orthostatic blood pressure measurement to ensure neither volume depletion nor volume overload.

6. All patients will undergo daily laboratory monitoring according to standard post-transplant care, including fasting blood sugar (FBS), BUN, Cr, electrolytes, and CBC. Calcium (Ca), phosphate (P), and magnesium (Mg) will be assessed every Monday, Wednesday, and Friday.

7. On postoperative day 4, blood and urine samples will be collected for a cytokine panel. Three tubes of clotted blood and three tubes of heparinized blood (3 mL each) will be sent for measurement of TNF-α, IL-6, IFN-γ, IL-10. Urine NGAL will be collected on day 4.

8. Patients with serum bicarbonate (HCO_3^-) < 15 mEq/L will receive rescue therapy according to the pre-specified protocol:

Severe metabolic acidosis is defined as a serum bicarbonate level below 15 mEq/L. Patients meeting this threshold will receive tiered rescue therapy based on severity: oral sodium bicarbonate (300 mg, 3 to 4 tablets, three times daily) for serum bicarbonate levels between 12.0 and 14.9 mEq/L, and intravenous 7.5% sodium bicarbonate infusions (50 to 100 mL over 1 hour) for levels below 12.0 mEq/L. Arterial blood gases, serum ketones, lactate, and serum electrolytes will be closely monitored before and after intravenous infusions.

9. Throughout the study, both groups will receive standard immunosuppression for kidney transplant recipients, including basiliximab or anti-thymocyte globulin, methylprednisolone, tacrolimus, and mycophenolic acid.

Handling of Residual Biological Specimens

Any residual blood specimens will be destroyed in accordance with standard laboratory procedures immediately after the completion of the study.

Sample Size and Calculation

The sample size for this randomized controlled trial (RCT) was calculated using the standard formula for comparing two independent means.[24–26]

$$n = \frac{2\sigma^2(Z_{\alpha/2} + Z_{\beta})^2}{(\mu_1 - \mu_2)^2}$$

Where:

- n = Sample size required per group
- σ = Standard Deviation (assumed to be equal for both groups)
- $Z_{\alpha/2}$ = The Z-value corresponding to the Type I error (α)
- Z_{β} = The Z-value corresponding to the Type II error (β), where Power = $1 - \beta$
- $(\mu_1 - \mu_2)$ = The clinically meaningful difference (Effect Size)

The calculation was based on the study by Medeiros et al.,[27] in which the postoperative mean serum bicarbonate (HCO_3^-) was 17.5 mEq/L in the normal saline group and 20 mEq/L in the balanced crystalloid (Ringer's lactate or Plasma-Lyte) group.

Assumptions: Type I error (α) = 0.05; Type II error (β) = 0.20; statistical power (1 − β) = 80%; $Z_{0.025}$ = 1.96; $Z_{0.2}$ = 0.84; standard deviation (σ) = 5 mEq/L; mean difference ($\mu_1 - \mu_2$) = 2.5 mEq/L; calculated sample size n = 63 per group.

Therefore, 63 participants per group are required. After adjusting for an anticipated 10% exclusion rate, the total sample size is set at least 140 participants.

11. Data Collection

The investigator will collect data from participants undergoing kidney transplantation at King Chulalongkorn Memorial Hospital. Collected variables will include baseline characteristics (sex, age, comorbidities, cause of CKD, type and duration of prior renal replacement therapy), donor characteristics, human leukocyte antigen (HLA) mismatch, ABO incompatibility, ischemic time, and intraoperative blood pressure. Data will be entered into a Microsoft Excel database. Participants will be identified by sequential study number only, without linkage to personal identifiers such as name or hospital number (HN), in order to prevent any inadvertent disclosure of patient information. All data will be entered solely by the investigator, stored on a password-protected computer, and accessible only to the investigator and the research advisor.

12. Planned Data and Statistics Analyses

- Continuous variables will be expressed as mean ± standard deviation (SD) or median with interquartile range (IQR), depending on data distribution. Between-group comparisons will be performed using the Student's t-test or the Mann–Whitney U test as appropriate.
- Categorical variables will be presented as frequencies and percentages and compared between groups using the chi-square (χ^2) test.
- Multilevel mixed-effects linear regression models will be utilized to account for repeated measures within individual participants.
- The primary and secondary outcomes will be evaluated utilizing a modified intention-to-treat analysis framework. To ensure that the physiological and metabolic effects of the assigned intravenous fluid regimens can be adequately assessed, a pre-specified post-randomization exclusion criterion will be applied. Specifically, patients who receive a total cumulative study fluid volume of less than 2 liters prior to the discontinuation of intravenous fluids will be excluded from the final analysis.
- Pre-specified subgroup analyses will be performed according to the stratification factors: living donor kidney transplantation (LDKT) versus deceased donor kidney transplantation (DDKT), immunological risk, and total IV fluid volume received.

13. Ethical Considerations

This study will adhere to the three fundamental principles of research ethics involving human subjects:

1) Respect for Persons

Written informed consent will be obtained from every participant. Complete information regarding the study will be provided to ensure full understanding before any independent decision to participate.

2) Beneficence / Non-maleficence

Participants will receive a clear explanation of the potential benefits and risks of study participation. Confidentiality will be maintained. Hospital numbers (HN) will not be used to link data to individuals. All study data and medical histories will be kept confidential, and participants' rights will be strictly protected. Findings will be reported only in aggregate form; individual data will not be disclosed.

3) Justice

The inclusion and exclusion criteria are clearly defined. All eligible patients undergoing kidney transplantation will be considered equally, without discrimination. Risks and benefits will be distributed equitably through random allocation to the study groups.

14. Expected or Anticipated Benefits

If this study demonstrates that Ringer's lactate solution produces less metabolic acidosis than the current regimen of normal saline plus 5% dextrose, the findings will support the transition from a two-fluid protocol to a single-fluid protocol. Such a change would reduce resource utilization and nursing workload and simplify fluid management in the first postoperative week. The study will additionally generate evidence on the impact of post-transplant IV fluid choice on metabolic acidosis, a contributor to delayed graft function (DGF), and may serve as the foundation for subsequent studies examining DGF and allograft rejection. These future efforts are anticipated to improve post-transplant care and ultimately extend allograft survival.

15. Challenges

Certain IV medications must be reconstituted in normal saline or 5% dextrose in water (5%DW)—for example, the immunosuppressant basiliximab and several antimicrobial agents. To mitigate this confounding effect, the type and volume of all fluids and all diluents used for medication reconstitution will be recorded. Specifically, 5%DW will be designated as the diluent for all medications to avoid contaminating the Ringer's lactate group with sodium chloride (NaCl).

Volume assessment based on IV fluid monitoring may be less accurate than central venous pressure (CVP) measurement. To address this, daily weight, inferior vena cava (IVC)

ultrasonography, and orthostatic blood pressure measurements will be used to confirm adequate volume status.

16. Risks and Investigator's Responsibility

Participants may experience minor risks associated with the additional blood draw performed specifically for the study on postoperative day 4 for cytokine measurement. These risks include pain, bruising, hematoma at the venipuncture site, and a small risk of infection. The risk of infection is very low and will be minimized by using aseptic phlebotomy technique. If venipuncture is unsuccessful after three attempts in any given patient, the investigator and attending physician will be notified, and cytokine sampling for that patient may be omitted.

Should pain, bruising, or hematoma develop at the venipuncture site, participants or their relatives should immediately inform the physician, nurse, or ward staff. First aid, such as cold compresses, will be administered promptly. Participants and their families will be informed that these events are foreseeable and not serious, and nursing staff will be instructed to exercise additional caution during subsequent blood draws.

17. Project Duration (Timeline)

Two (2) years.

18. Venue of the Study

King Chulalongkorn Memorial Hospital, Bangkok, Thailand.

19. References

1. Gilbert J, Lovibond K, Mooney A, Dudley J, Guideline C. Renal replacement therapy: summary of NICE guidance. BMJ. 2018;363:k4303.

2. Wang JH, Skeans MA, Israni AK. Current status of kidney transplant outcomes: dying to survive. Adv Chronic Kidney Dis. 2016;23:281–6.

3. Wu WK, Famure O, Li Y, Kim SJ. Delayed graft function and the risk of acute rejection in the modern era of kidney transplantation. Kidney Int. 2015 Oct;88(4):851–8.

4. Ponticelli C, Reggiani F, Moroni G. Delayed graft function in kidney transplant: risk factors, consequences and prevention strategies. J Pers Med. 2022;12(10):1557.

5. Perico N, Cattaneo D, Sayegh MH, Remuzzi G. Delayed graft function in kidney transplantation. Lancet. 2004;364:1814–27.

6. Yarlagadda SG, Coca SG, Formica RN Jr, Poggio ED, Parikh CR. Association between delayed graft function and allograft and patient survival: a systematic review and meta-analysis. Nephrol Dial Transplant. 2009;24:1039–47.

7. Liu X, Lu M. Normal saline: past, present, and future. Sci Prog. 2023;106(2).

8. Lobo DN, Awad S. Should chloride-rich crystalloids remain the mainstay of fluid resuscitation to prevent 'pre-renal' acute kidney injury?: con. Kidney Int. 2014 Dec;86(6):1096–105.

9. Kraut JA, Madias NE. Metabolic acidosis: pathophysiology, diagnosis and management. Nat Rev Nephrol. 2010;6:274–85.

10. Semler MW, Self WH, Wanderer JP, et al.; SMART Investigators and the Pragmatic Critical Care Research Group. Balanced crystalloids versus saline in critically ill adults. N Engl J Med. 2018 Mar 1;378(9):829–39.

11. Self WH, Semler MW, Wanderer JP, et al.; SALT-ED Investigators. Balanced crystalloids versus saline in noncritically ill adults. N Engl J Med. 2018 Mar 1;378(9):819–28.

12. Yunos NM, Bellomo R, Hegarty C, Story D, Ho L, Bailey M. Association between a chloride-liberal vs chloride-restrictive intravenous fluid administration strategy and kidney injury in critically ill adults. JAMA. 2012;308:1566–72.

13. O'Malley CMN, Frumento RJ, Hardy MA, Benvenisty AI, Brentjens TE, Mercer JS, Bennett-Guerrero E. A randomized, double-blind comparison of lactated Ringer's solution and 0.9% NaCl during renal transplantation. Anesth Analg. 2005;100(5):1518–24.

14. Wan S, Roberts MA, Mount P. Normal saline versus lower-chloride solutions for kidney transplantation. Cochrane Database Syst Rev. 2016;(8):CD010741.

15. Saini V, Samra T, Naik BN, Ganesh V, Garg K, Sethi S, et al. Normal saline versus balanced crystalloids in renal transplant surgery: a double-blind randomized controlled study. Cureus. 2021;13(9):e18247.

16. Collins MG, Fahim MA, Pascoe EM, Hawley CM, Johnson DW, Varghese J, et al. Balanced crystalloid solution versus saline in deceased donor kidney transplantation (BEST-Fluids): a pragmatic, double-blind, randomised, controlled trial. Lancet. 2023;402(10396):105–17.

17. Etezadi F, Pourfakhr P, Mojtahedzade M, et al. Effects of tight versus non-tight control of metabolic acidosis on early renal function after kidney transplantation. DARU J Pharm Sci. 2012;20:36.

18. Tejchman K, Domanski L, Sienko J, Sulikowski T, Kaminski M, Romanowski M, et al. Early acid-base balance disorders during kidney transplantation. Transplant Proc. 2006 Jan–Feb;38(1):123–6.

19. Ponticelli C, Reggiani F, Moroni G. Delayed graft function in kidney transplant: risk factors, consequences and prevention strategies. J Pers Med. 2022 Sep 21;12(10):1557.

20. Knechtle SJ, Marson LP, Morris PJ. Kidney Transplantation. 8th ed. Elsevier; 2020.

21. Bellomo R, Kellum JA, Ronco C. Acute kidney injury. Lancet. 2012 Aug 25;380(9843):756–66.

22. Lee DW, Faubel S, Edelstein CL. Cytokines in acute kidney injury (AKI). Clin Nephrol. 2011 Sep;76(3):165–73.

23. Jaber S, Paugam C, Futier E, Lefrant JY, Lasocki S, Lescot T, et al.; BICAR-ICU Study Group. Sodium bicarbonate therapy for patients with severe metabolic acidaemia in the intensive care unit (BICAR-ICU): a multicentre, open-label, randomised controlled, phase 3 trial. Lancet. 2018 Jul 7;392(10141):31–40.

24. Ngamjarus C, Pattanittum P. n4Studies: application for sample size calculation in health science research. Version 2.3. App Store; 2024.

25. Rosner B. Fundamentals of Biostatistics. 5th ed. Duxbury: Thomson Learning; 2000. p. 308.

26. Ngamjarus C. Sample Size Calculation for Health Science Research. 1st ed. Khon Kaen, Thailand: Khon Kaen University Printing House; 2021.

27. Medeiros H, Lima PH, Souza Junior VS, Souza DA, Pinheiro AM, Martins RR, et al. A comparison between saline and balanced solutions in kidney transplants: a randomized clinical trial. Cureus. 2023 Dec 1;15(12):e49813.

28. Hayes WN, Laing E, Brown R, Silsby L, Smith L, Thomas H, et al. A pragmatic, open-label, randomized controlled trial of Plasma-Lyte-148 versus standard intravenous fluids in children receiving kidney transplants (PLUTO). Kidney Int. 2024 Feb;105(2):364–75.
